# Multi-site cytokine levels are predictive of primary graft dysfunction following lung transplantation

**DOI:** 10.3389/ti.2026.15874

**Published:** 2026-09-14

**Authors:** Dianna Nord, Jason Cory Brunson, Logan Langerude, Hassan Moussa, Blake Gill, Mindaugas Rackauskas, Ashish Sharma, Christine Lin, Amir Emtiazjoo, Carl Atkinson

**Affiliations:** 1 Division of Pulmonary, Critical Care, and Sleep Medicine, University of Florida, Gainesville, FL, United States; 2 Laboratory for Systems Medicine, University of Florida, Gainesville, FL, United States; 3 Department of Surgery, Comprehensive Transplant Center, Feinberg School of Medicine, Northwestern University Chicago, Chicago, IL, United States; 4 Department of Surgery, University of Florida, Gainesville, FL, United States; 5 Department of Medicine, University of California San Diego, San Diego, CA, United States

**Keywords:** cytokines, lung transplantation, machine learning, predictive models, primary graft dysfunction

## Abstract

There is an urgent need to better understand the pathophysiology of primary graft dysfunction (PGD) to develop point-of-care methods predicting those at risk. We utilized a multiplex multivariable approach to define cytokines, chemokines, and growth factors in patient-matched biospecimens to identify factors predictive of PGD. Biospecimens were collected from patients undergoing bilateral lung transplantation (LTx) from three sites: donor lung perfusate, post-transplant bronchoalveolar lavage (BAL) fluid (2h), and plasma (2h, 24h, 72h, and 1 and 2 wks). A 71-multiplex panel was performed on each. Cross-validated logistic regression (LR) and random forest (RF) models determined whether analytes from each site, alone or combined with clinical data, discriminated PGD grade 0 (n = 9) vs. 3 (n = 8). BAL fluid at 2h was most predictive of PGD (LR, 0.825; RF, 0.919), followed by multi-timepoint plasma (LR, 0.841; RF, 0.653), then perfusate (LR, 0.565; RF, 0.448). Combined clinical, BAL, and plasma data yielded the strongest performance (LR, 1.000; RF, 1.000). BAL collected 2h post-transplant showed the strongest discriminatory signal for severe PGD in this exploratory cohort. This integrative approach identified IL-1RA, BCA-1, and Fractalkine as hypothesis-generating candidate biomarkers that warrant validation in larger independent studies.

## Graphical Abstract

**Figure:**
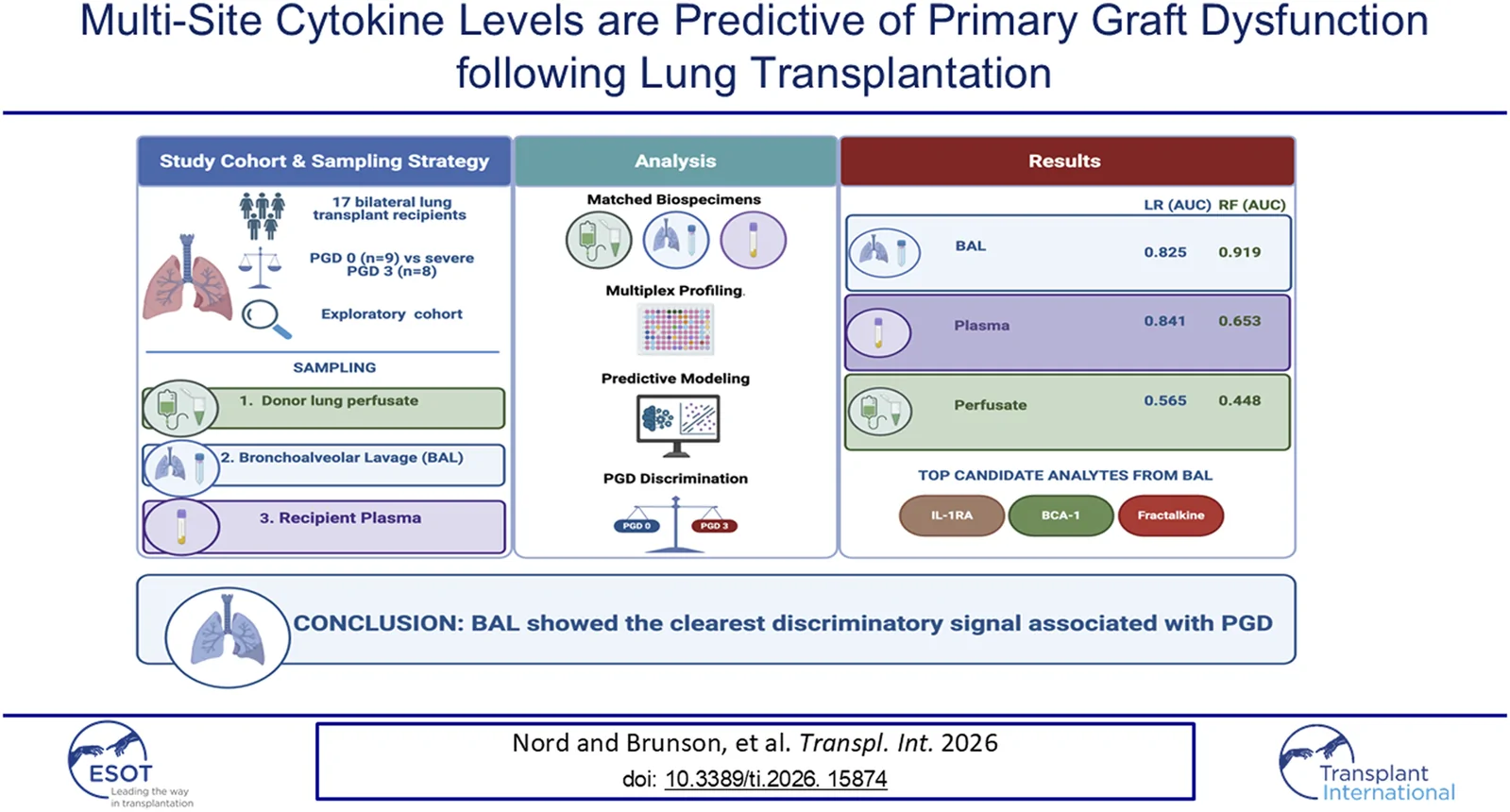

## Introduction

Lung transplantation (LTx) is an acceptable therapy for end-stage lung disease. However, successful post-transplantation outcomes are significantly impacted by the development of severe primary graft dysfunction (PGD), which is associated with early mortality and a more rapid onset of chronic lung allograft dysfunction (CLAD) [1–4]. As such, there is an urgent need to better understand PGD pathobiology and identify those at enhanced risk. The pathophysiology of PGD is complex and involves a multifactorial inflammatory process that likely starts in the donor lung and is exacerbated by ischemia reperfusion injury (IRI). Given the contribution of inflammation in PGD, several studies have leveraged the presence of pro-inflammatory cytokines as prognostic biomarkers. These studies have largely measured the presence of soluble factors in recipient plasma, with elevated levels of plasma cytokines such as IL-6, IL-8, IP-10, MCP-1, and endothelial/epithelial injury markers shown to be associated with severe PGD [5–13].

Though plasma provides a valuable window into the biological/inflammatory processes detected on a systemic level, a handful of studies have analyzed bronchoalveolar lavage (BAL) fluid as a more representative sample of the transplanted lung-specific microenvironment. Elevated BAL levels of IL-6, IL-8, and Eotaxin were shown to be associated with PGD [14–16]. Further, *ex vivo* lung perfusion (EVLP) studies have shown that elevated IL-8, IL-6, and GRO-α levels in perfusion solution are associated with poorer graft outcomes [17, 18].

Together, these studies show the potential value of measuring inflammatory and injury markers as predictors of PGD. Most factors identified to date have been selected for investigation by association, in that they have been previously implicated in other acute lung injuries and then subsequently investigated in LTx. While these studies have guided our understanding of PGD, their hypothesis-driven focus may limit the discovery of other mediators or predictors of PGD. Thus, here we utilized a 71-analyte multiplex approach composed of a broad array of cytokine, chemokines, and growth factors in concert with matched donor perfusate, recipient plasma, and BAL to identify novel biomarkers and/or mechanistic factors associated with PGD. By examining multiple analytes across diverse samples, our study aimed to mitigate selection bias and uncover new correlates to PGD. We further hypothesized that a machine learning approach may serve as a valuable tool that complements classical statistics for biomarker discovery. Here we demonstrate that cytokine expression in BAL collected 2h post-LTx best predicts PGD development. Further, using a model reduction process, we identified the three strongest BAL predictors of PGD development were BCA-1 (CXCL13), Fractalkine, and IL-1Ra, factors that have not been extensively studied in the context of PGD. Together, our results provide a methodological foundation for further development of predictive modeling that could result in the discovery of analytes that can be used in a clinically relevant point-of-care test and provide novel targets for PGD mechanistic studies.

## Methods and materials

### Inclusion/exclusion criteria

Samples were acquired from the University of Florida Transplant Tissue Bank from patients undergoing a first-time bilateral lung transplant between May 2021 and May 2022. Nineteen patients who had a complete sample set and had the same PGD grade at all time points, either 0 (*n* = 10) or 3 (*n* = 9), were selected. PGD was graded as described [19]^.^ To maximize phenotypic contrast within a limited cohort, the primary analyses were restricted to recipients with persistently absent PGD (grade 0 at all assessed time points) or persistently severe PGD (grade 3 at all assessed time points). This design was selected to reduce ambiguity introduced by transient, intermediate, or fluctuating PGD grades and improve the likelihood of detecting biologically meaningful signals in an exploratory setting. For accuracy and consistency, transplant recipients without severe graft dysfunction are referred to throughout as PGD0 or PGD– recipients, whereas patients with persistently severe PGD, will be termed PGD+. Donor and recipient demographics for each group are detailed in Table 1. This study was approved by the University of Florida Institutional Review Board IRB#202200536.

**TABLE 1:** Baseline donor and recipient characteristics.

Donor Characteristics	PGD– (n = 9)	PGD+ (n = 8)	p-value
Median donor age	37 (16, 60)	37 (18, 60)	.902
Donor sex			
Male	89% (8)	25% (2)	**.020**
Female	11% (1)	75% (6)	​
Donor race/Ethnicity			
White	44% (4)	50% (4)	–
Hispanic/Latino	33% (3)	38% (3)	​
Black or african american	22% (2)	–	​
American indian	–	13% (1)	​
Static storage Standard 4 °C	100% (9)	50% (4)	**.033**
10 °C	–	50% (4)	​
Smoking history			
Yes	56% (5)	38% (3)	.637
No	44% (4)	63% (5)	​
Heavy alcohol use history			
Yes	22% (2)	13% (1)	.999
No	78% (7)	88% (7)	​

Recipient characteristics	PGD– (n = 9)	PGD+ (n = 8)	p-value
Median recipient age	61 (48, 74)	59 (35, 73)	.418
Mean recipient BMI at listing	24.9	26.9	.256
Recipient sex Male	78% (7)	13% (1)	.015
Female	22% (2)	88% (7)	​
UNOS lung disease diagnosis			
Group			
Group A: Obstructive lung disease	3	0	–
Group B: Pulmonary vascular	1	0	​
Disease			
Group C: Cystic fibrosis	0	0	
Group D: Restrictive lung disease	5	9	​
Mean LAS Score	47.9 (33.1, 92.4)	57.1 (36.5, 93.3)	.462
% ECMO pre-Tx	10% (1)	22% (2)	.577
% ECMO post-Tx	10% (1)	67% (5)	.013
% Sensitization protocol	– (0)	50% (4)	.023

Donor and Recipient characteristics by primary graft dysfunction status. Differences between groups in categorial variables were analyzed by Fisher’s Exact Comparison and differences in numerical variables were analyzed by unpaired Student’s t-test, with unadjusted p-values p < 0.05 Abbreviations. PGD–: Primary Graft Dysfunction grade 0 at all time points; PGD+: Primary Graft Dysfunction grade 3 at all time points; BMI: Body Mass Index; UNOS: United Network for Organ Sharing, LAS: Lung Allocation Score, ECMO: Extracorporeal Membrane Oxygenation.

### Sample collection

We analyzed three distinct sample types: (1) plasma, (2) bronchoalveolar lavage fluid (BAL), and (3) donor lung perfusate solution. Plasma was processed from whole blood collected 12h prior to transplant (pre) and at 2h, 24h, 72h, 1wk, and 2wk post-LTx. BAL fluid was obtained following reperfusion, at a time point matched to the 2h blood sample, “T0”. BAL was collected following the ISHLT recommendations [20]. Donor lung Perfadex® (Xvivo, Sweden) perfusate solution was obtained by collecting the initial volume of 2–3 mL of preservation solution passed out of the lung vasculature at the time of reperfusion. All processed samples were stored at −80 °C until time of analysis.

### Multiplex assays

We utilized Eve Technologies’ Cytokine, Chemokine, Growth Factor 71-Plex Clinical RUO Test (HD71-CLIN). Samples were run on Luminex 100/200™ instruments (Luminex/DiaSorin, Saluggia, Italy), with Bio-Plex Manager™ (BPM) software (BioRad, Hercules, CA) by Eve Technologies (Calgary, Canada), a Clinical Laboratory Improvement Amendments (CLIA)-approved facility. The full analyte list, with analyte abbreviations and assay detection limits, is listed in Supplementary Tables 1A, B. Since a relative absence (or low levels) of any particular analyte could be meaningful with respect to patterns of cytokine expression, any sample with a fluorescence value below the range of its standard curve was included in the analysis by assigning a concentration value corresponding to the minimum sample value for the same analyte. We recognize that this approach may introduce bias. Therefore, we performed prespecified robustness analyses using alternative preprocessing strategies: first, repeating analyses without concentration imputation, and second, analyzing fluorescence intensity values directly, for which missingness was minimal (see robustness checks section).

### Statistical, computational analysis software, and preprocessing

Computations were performed in R [21], using Tidyverse and Tidymodels workflows [22, 23] and additional packages for specific analyses [24–35]*.* Smoking and alcohol history were collapsed to logicals. We offset log-transformed concentrations before analysis. As this study was designed as an exploratory biomarker discovery analysis, our objective was to identify candidate inflammatory mediators and specimen types associated with severe PGD rather than to test a small number of prespecified hypotheses. Accordingly, p-values from univariable screening analyses were not adjusted for multiple comparisons and should be interpreted as descriptive signals intended to prioritize candidates for further validation rather than as definitive evidence of association. All findings, particularly those emerging from high-dimensional multiplex and modeling analyses, should therefore be considered hypothesis-generating.

### Protein concentrations and pairwise associations

We visualized protein co-concentration patterns within each medium using dendrogram-annotated heatmaps [27] and correlation plots [28]. We used Kruskal–Wallis (KW) tests to identify proteins whose plasma concentrations changed over time. We included these in a principal components analysis (PCA) and generated a biplot with an average trajectory represented by segments between consecutive centroids.

### Discrimination of PGD

KW (perfusate, BAL fluid) and multivariate Cramér [36] (plasma at all time points) tests were used to identify proteins that discriminated between the PGD– and PGD+ subgroups. Log-concentrations between the subgroups were visualized using jitter and line plots. Biplots of plasma concentrations were also generated with PGD-stratified average trajectories and are reported in the Methodological Supplement (M1).

### Prediction of PGD

Cross-validated logistic regression (LR), random forest (RF), and nearest neighbors (NN) models were used to determine whether several sets of predictors could discriminate between PGD– and PGD+. To better reflect practice conditions, no missing values were imputed for predictive analysis; analytes with several missing values were excluded. The pre-processing steps are detailed in the Methodological Supplement. Model families were optimized over hyperparameter grids. For each model specification, the area under the receiver operating characteristic curve (AUROC) was calculated from stratified leave-one-out cross-validation, in which each test set comprised one PGD– and one PGD+ case. We repeated this procedure 12 times to ensure diversity in the test sets.

The predictive value of data from each site, namely perfusate, BAL fluid, plasma (pre-Tx, T0, and 24h), and clinic (donor, recipient, and perioperative), was assessed. We ranked the model specifications by mean AUROC. Results were summarized using line–range plots and tabulated optimal and near-optimal performance by model family using each combination of sites. Clinical variables were incorporated as a separate predictor set consisting of available donor, recipient, and perioperative characteristics contained within the de-identified tissue bank dataset.

We next fitted optimized model specifications to complete data from each combination of sites and used absolute coefficient estimates (LR) and mean decrease in accuracy (RF) to rank predictors. To assess the potential feasibility of a point-of-care test, we included the top predictors from both families in a LR LASSO, using data from the site that yielded the best predictions.

### Robustness checks

We tested the robustness of our results by re-running the full analysis after each of two alternative pre-processing choices: (1) for exploratory analysis, not imputing out-of-range values and, for predictive analysis, imputing; and (2) using fluorescence intensities (FIs), for which very few values were missing, rather than concentrations.

## Results

### Sample characteristics and group differences

Seventeen patients with complete data were included in the analysis (PGD–, *n* = 9; PGD+, *n* = 8). Consistent with published risk factors, PGD+ patients were predominantly female and pre-sensitized (Table 1).

### Plasma protein concentrations and pairwise comparisons

We observed consistent protein clustering based on concentrations of analytes in plasma over the first three time points (pre-Tx, T0, and 24h) (Figure 1A). One outlier case (G) had higher levels in low-concentration clusters. With six exceptions, panels clustered by time point. Next, we selected a distance threshold, based on visual inspection of dendrograms to identify clusters at T0 (Figure 1B). There was one singleton, IL-1RA, the only analyte to be detectably negatively correlated with others (TARC and VEGF-A); a small cluster containing its negative correlates with ENA-78, PDGF-AA, PDGF-AB/BB, MDC, CTACK, RANTES, and Eotaxin-2; another small cluster comprising TRAIL, MCP-2, MCP-1, G-CSF, and IL-6; and the remaining proteins in one large cluster.

**FIGURE 1:**
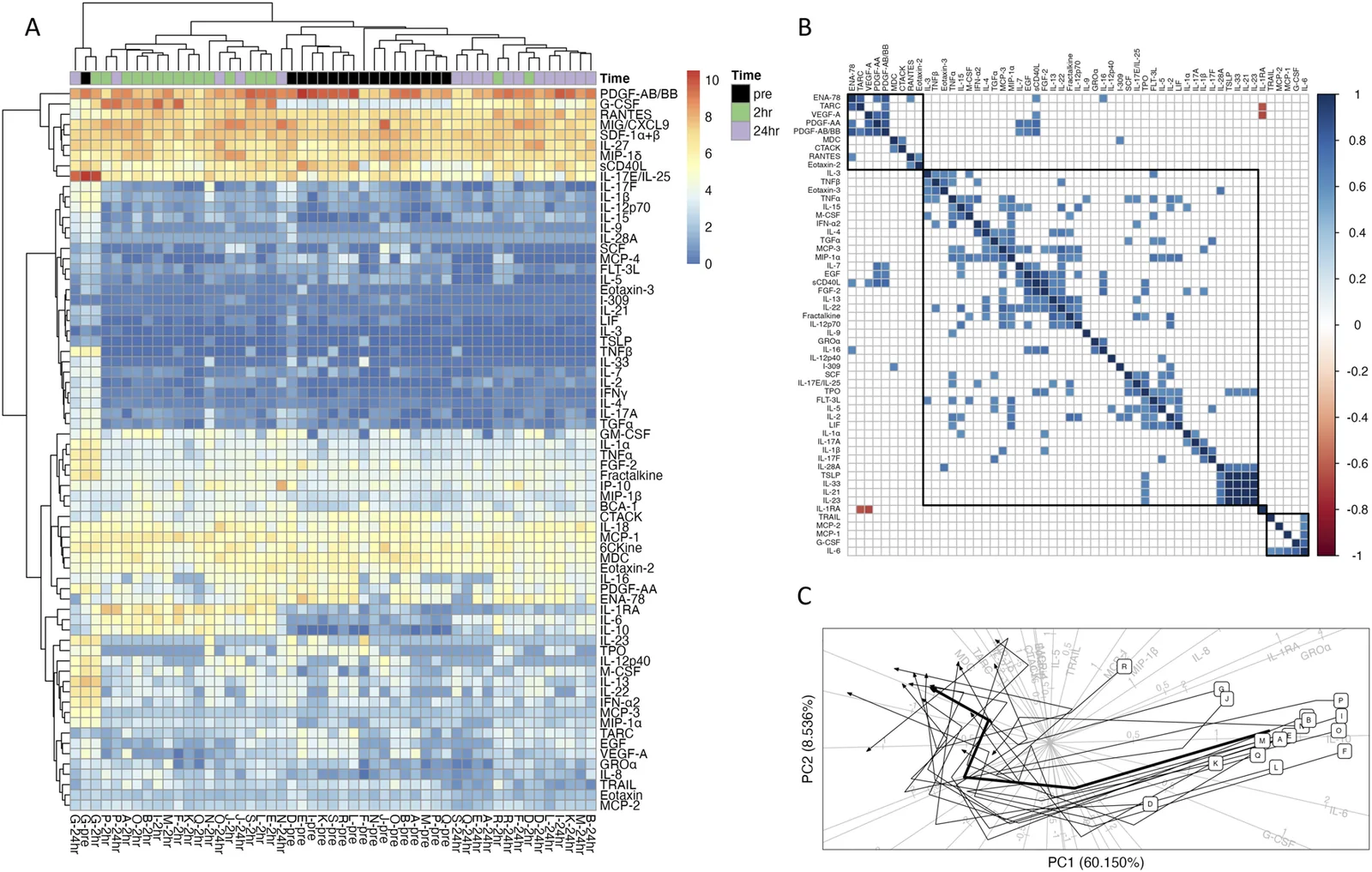
Log-concentrations of 71 proteins in plasma at near-transplantation time points. Protein levels and their changes during the peri-operative period were similar across all but one subject. **(A)** Clustered heatmap for all subjects at pre-Tx, T0, and 24h time points, using complete-linkage clustering on cases and proteins. Blue (red) indicates lower (higher) levels. **(B)** Correlation matrix of soluble factors measured in plasma from all subjects at T0. Only correlations with unadjusted p < 0.01 in Spearman tests are filled. Proteins are ordered by complete-linkage clustering in order to juxtapose more correlated proteins. Note that complete-linkage clustering is heuristic, so the order may differ from that of **(A)**. **(C)** Principle component analysis (PCA) biplot of time-discriminating proteins at all subject–time points. Values at pre-Tx are labeled by unique keys. Segments connect adjacent time points for each subject. Segments are grouped by primary graft dysfunction (PGD) status. Thickened segments connect time point centroids, stratified by PGD status.

The PCA distinguished two dimensions of variation in plasma concentrations over time (Figure 1C). IL-1RA, GROα, IL-10, IL-6, and G-CSF loaded strongly onto PC1, all positively. From pre-Tx to 24h, PC1 decreased, then remained low through 2wk. MDC, TARC, IL-3, MIP-1δ, CTACK, Eotaxin, IL-2, IL-5, and TRAIL loaded strongly and negatively onto PC2, MDC, and TARC. PC2 decreased from pre-Tx to T0 then increased gradually through 2wk. MCP-1, MIP-1β, and IL-8 correlated more with each other than with either PC. The average trajectory reflected these patterns.

### Plasma protein discrimination of PGD

We next analyzed whether concentration changes in plasma differed by PGD status. Analysis identified two-to-four clusters at T0, which broadly agree between the PGD– and PGD+ subgroups (Figure 2). Mean trajectories did not differ systematically between the PGD– and PGD+ groups (Supplementary Figure M1). We did find evidence of systematically different concentrations of certain proteins at some time points (Table 2), notably BCA-1 at 1wk and 6CKine at T0. We detected only two proteins with different trajectories (Table 3; Figure 3): BCA-1 and IL-22 (p < 0.05), both with higher concentrations in the PGD+ group, but otherwise similar trajectories. Several proteins (MCP-1, MIP-1β, IL-6, G-CSF, GROα, IL-8, IL-10, and IL-1RA) associated with PC1 spiked at T0 and then regressed toward their pre-LTx levels in both groups (Figure 3).

**FIGURE 2:**
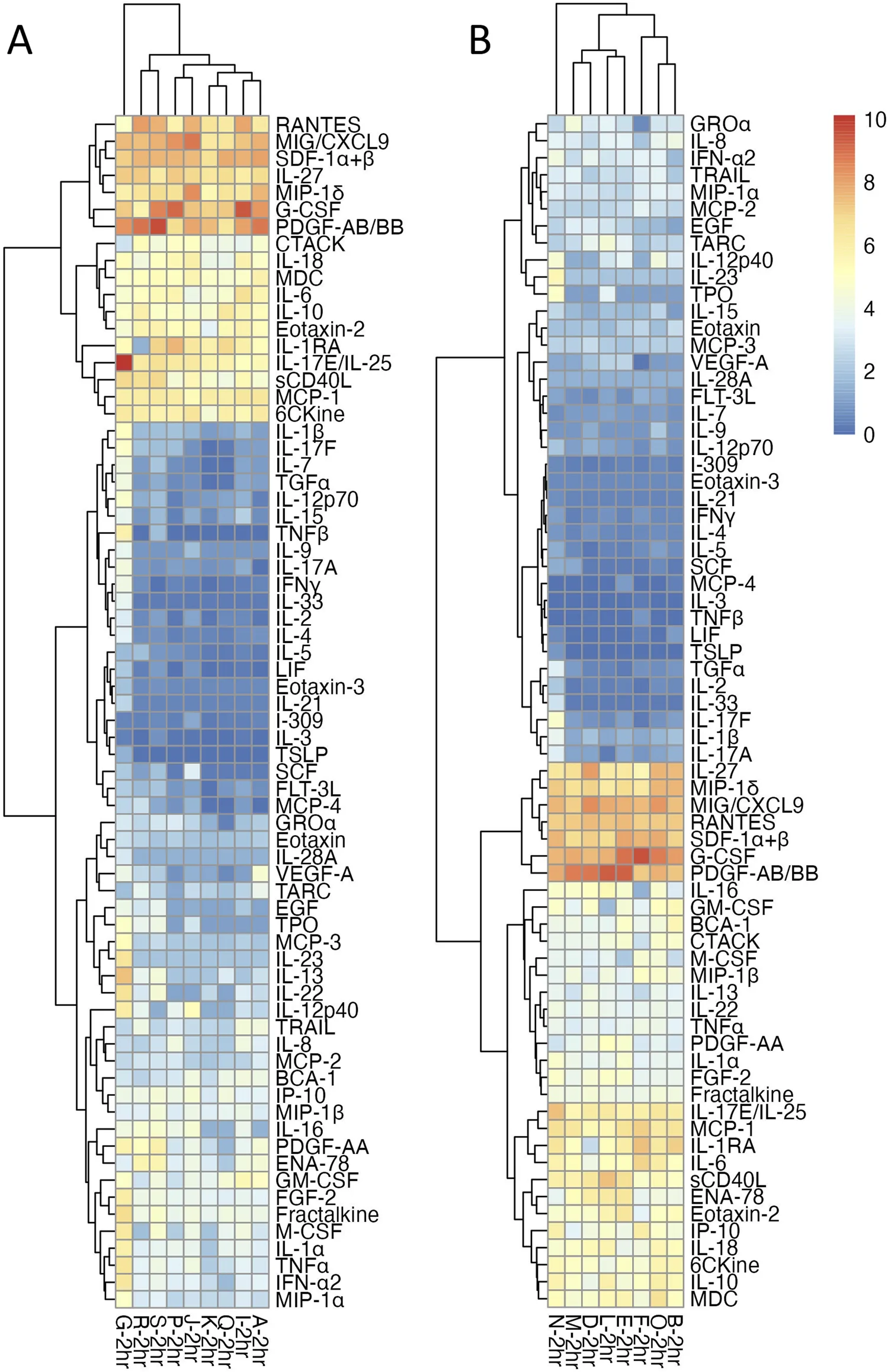
Differences in protein log-concentrations in plasma by primary graft dysfunction (PGD) grade. Clustered heatmaps **(A)** for PGD– and **(B)** for PGD+ subjects.

**TABLE 2:** Results of Kolmogorov–Smirnov tests of differences between undetected and severe primary graft dysfunction cases in protein concentrations in plasma at single time points.

Time	Protein	*n*_0_	*n*_3_	Statistic	p-value
pre	BCA-1	9	8	0.667	0.02024
2h	6CKine	9	8	0.778	0.00255
2h	IL-22	9	8	0.778	0.00428
2h	IL-16	9	8	0.750	0.01119
2h	MCP-4	9	8	0.542	0.04977
24h	6CKine	9	8	0.778	0.00428
24h	IL-6	9	8	0.764	0.00831
24h	IL-22	9	8	0.667	0.02024
24h	MCP-1	9	8	0.667	0.02024
24h	IL-15	9	8	0.639	0.04689
24h	BCA-1	9	8	0.639	0.04689
72h	I-309	9	8	0.764	0.00831
72h	PDGF-AA	9	8	0.667	0.02024
72h	IL-12p40	9	8	0.556	0.02941
72h	BCA-1	9	8	0.653	0.03357
72h	IL-3	9	8	0.556	0.04689
72h	IL-6	9	8	0.639	0.04689
1wk	BCA-1	9	8	0.875	0.00140
1wk	TRAIL	9	8	0.750	0.01119
1wk	PDGF-AB/BB	9	8	0.653	0.03357
1wk	MIP-1δ	9	8	0.653	0.03357

Analytes are listed by sample time point first and in order of statistical evidence, up to unadjusted p < 0.05. See Supplementary Table 1 for details on the full set of analytes. BCA-1, B-cell-attracting chemokine 1 (CXCL13); 6CKine, secondary lymphoid-tissue chemokine (CCL21); IL-22, interleukin-22; IL-16, interleukin-16; MCP-4, monocyte chemoattractant protein 4 (CCL13); IL-6, interleukin-6; MCP-1, monocyte chemoattractant protein 1 (CCL2); IL-15, interleukin-15; I-309, T-lymphocyte-secreted protein I-309 (CCL1); PDGF-AA, platelet-derived growth factor-AA; IL-12p40, interleukin-12 p40 subunit; IL-3, interleukin-3; TRAIL, TNF-related apoptosis-inducing ligand; PDGF-AB/BB, platelet-derived growth factor-AB/BB; MIP-1δ, macrophage inflammatory protein-1 delta (CCL15).

**TABLE 3:** Results of multivariate Crámer tests of differences between undetected and severe primary graft dysfunction cases in protein concentrations across all time point**s**.

Protein	*n*_0_	*n*_3_	Statistic	p-value
BCA-1	42	42	5.007	0.0050
IL-22	42	42	4.876	0.0240
IL-15	42	42	1.646	0.0609
6CKine	42	42	1.892	0.0829
Eotaxin	42	42	0.850	0.0899

Analytes are listed in order of statistical evidence, up to unadjusted p < 0.1. See Supplementary Table 1 for details on the full set of analytes. Abbreviations; BCA-1, B-cell-attracting chemokine 1 (CXCL13); IL-22, interleukin-22; IL-15, interleukin-15; 6CKine, secondary lymphoid-tissue chemokine (CCL21); Eotaxin, eosinophil chemotactic protein 1 (CCL11).

**FIGURE 3:**
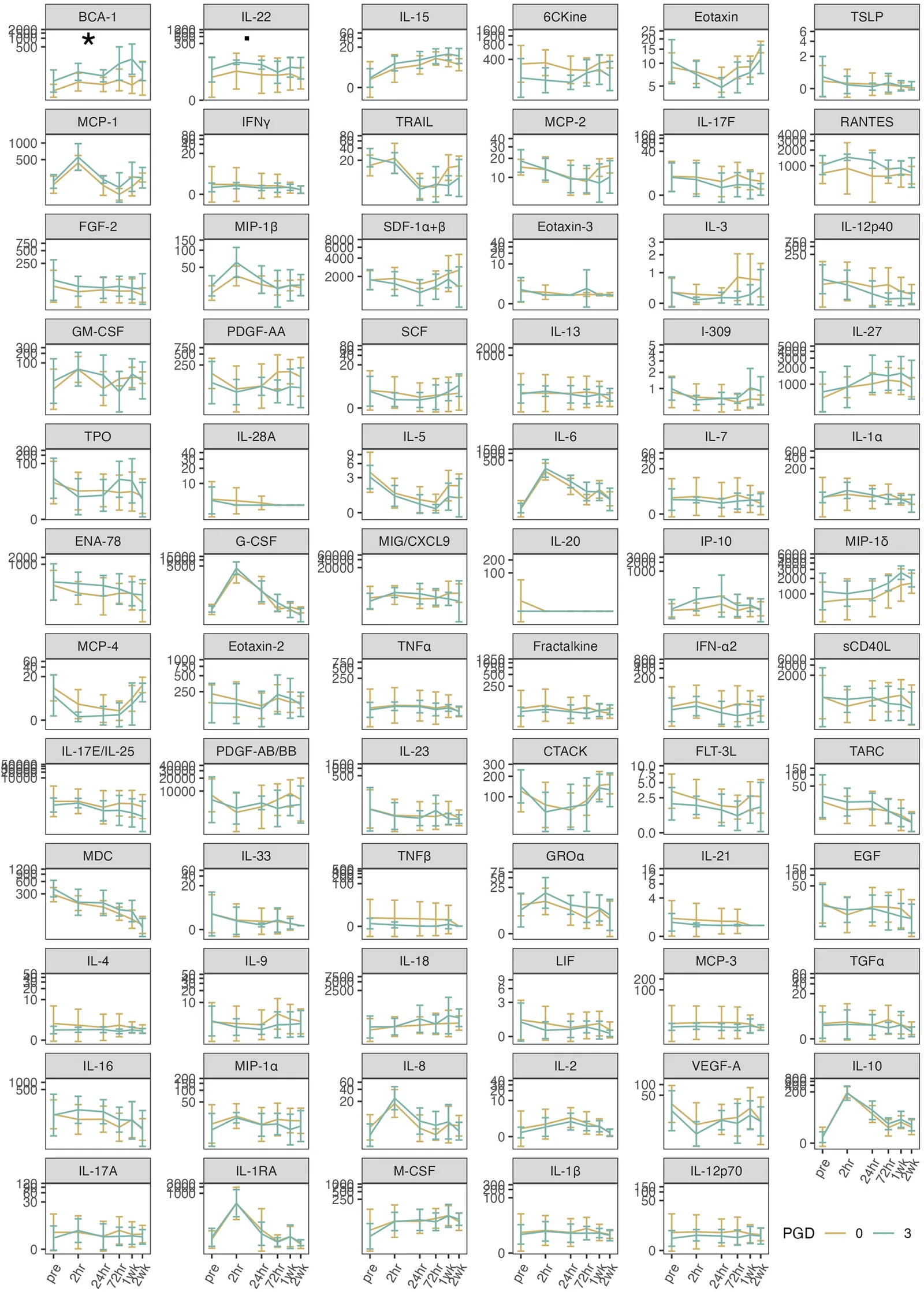
Differences in protein log-concentrations in plasma by PGD grade. Mean concentration and 1–standard deviation intervals across all time points, grouped by PGD grade. The panels are in order of increasing multivariate Cramér test unadjusted p-value and marked: ***p < 0.0001; **p < 0.001; *p < 0.01; p < 0.05. See the Methodological Supplement for a table of p-values. Note that tests detected differences in BCA-1 and IL-22 between PGD–and PGD+, though this number (2) is expected by chance. Irrespective of PGD grade, cytokines and chemokines peaked 24h post-transplantation in response to ischemia reperfusion injury.

### Perfusate protein discrimination of PGD

The same protein panel used for plasma analysis was used to assess perfusate solutions collected at the time of transplantation. Two proteins were consistently highly expressed in perfusate: FGF-2 and MIP-1δ. The remainder formed one higher- and one lower-concentration cluster (Figure 4A). The cases did not clearly cluster and three outliers were detected (S, P, and I). Clustering did not correlate with PGD grade. A direct comparison of all cases (Figure 4B) suggests that organs with higher analyte levels preceded PGD+, but we found no evidence that any single protein discriminated between PGD– and PGD+ (Figure 4C) (Table 4). Of 66 proteins with sufficient data, only two tests yielded p <0.01 (IL-17A and IL-1RA), a number expected by chance.

**FIGURE 4:**
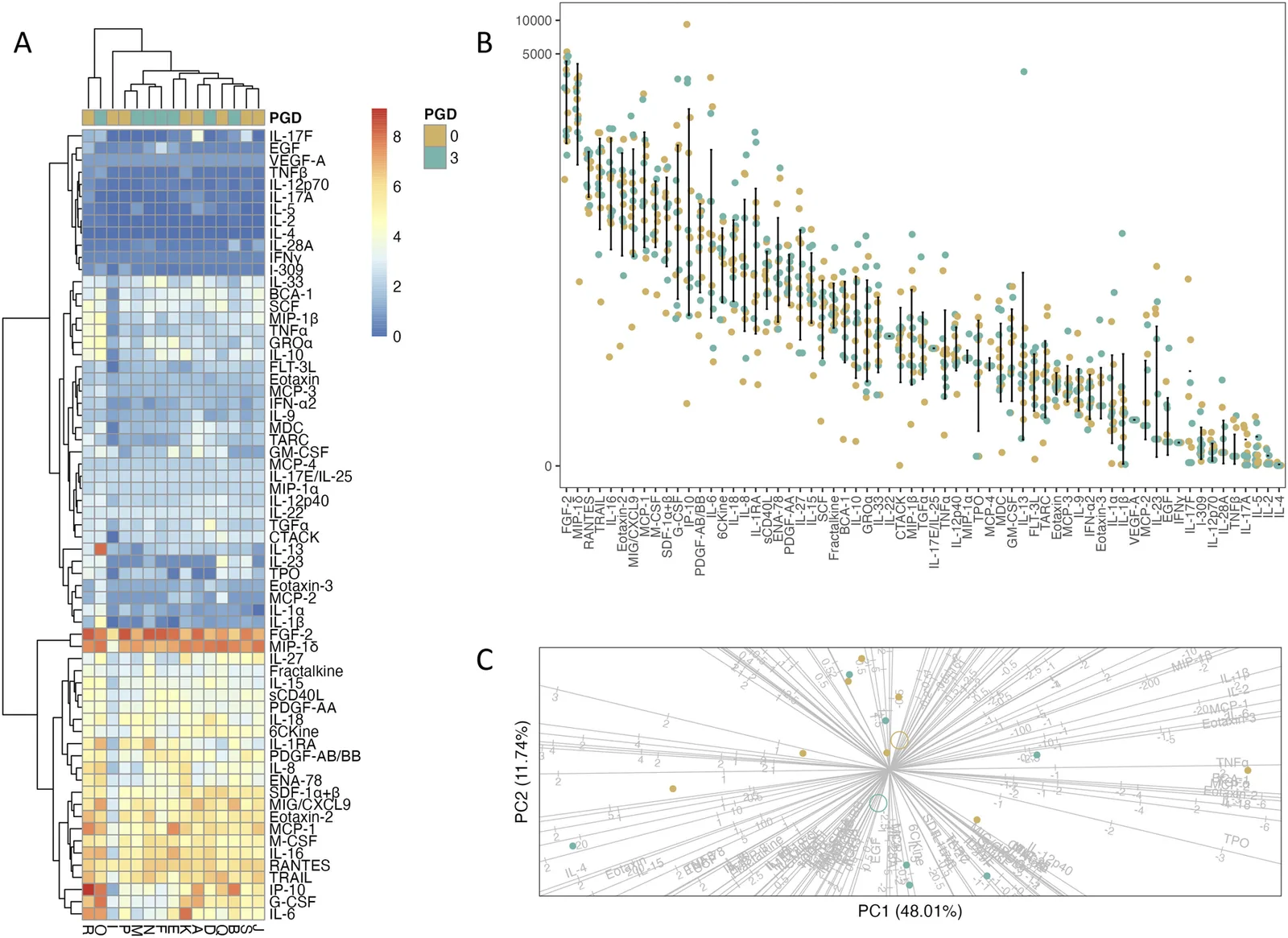
Differences in protein log-concentrations in perfusate by primary graft dysfunction (PGD) negative versus severe PGD 3. Protein levels were not detectably different between PGD–and PGD+ subjects. **(A)** Clustered heatmap for all subjects at T0, using complete-linkage clustering on cases and proteins, annotated by PGD grade. **(B)** Jitter plots, grouped by PGD grade. Proteins are in order of decreasing median concentration. **(C)** PCA biplot on all available protein concentrations. PGD group averages are indicated by open circles.

**TABLE 4:** Results of Kolmogorov–Smirnov tests of differences between undetected and severe primary graft dysfunction cases in protein concentrations in perfusate.

Protein	*n*_0_	*n*_3_	Statistic	p-value
IL-17A	8	7	0.625	0.0357
IL-1RA	8	7	0.607	0.0870

Analytes are listed in order of statistical evidence, up to unadjusted p < 0.1. See Supplementary Table 1 for details on the full set of analytes. Abbreviations; IL-17A, interleukin-17A; IL-1RA, interleukin-1 receptor antagonist.

### BAL protein discrimination of PGD

BAL also did not clearly cluster cases or discriminate PGD groups (Figure 5A), However, higher concentrations generally correspond to PGD+ (Figure 5B). This pattern was statistically evidenced for several proteins (Table 5): IL-1RA (*p <* 0.01), Fractalkine, BCA-1, Eotaxin, IL-12p40, IL-6, IP-10, and M-CSF (*p <* 0.05). Unlike the other sample types, PCA on all 71 proteins showed a clear separation between PGD– and PGD+ cases (Figure 5C), indicating that much of the variation in cytokine profiles was associated with PGD.

**FIGURE 5:**
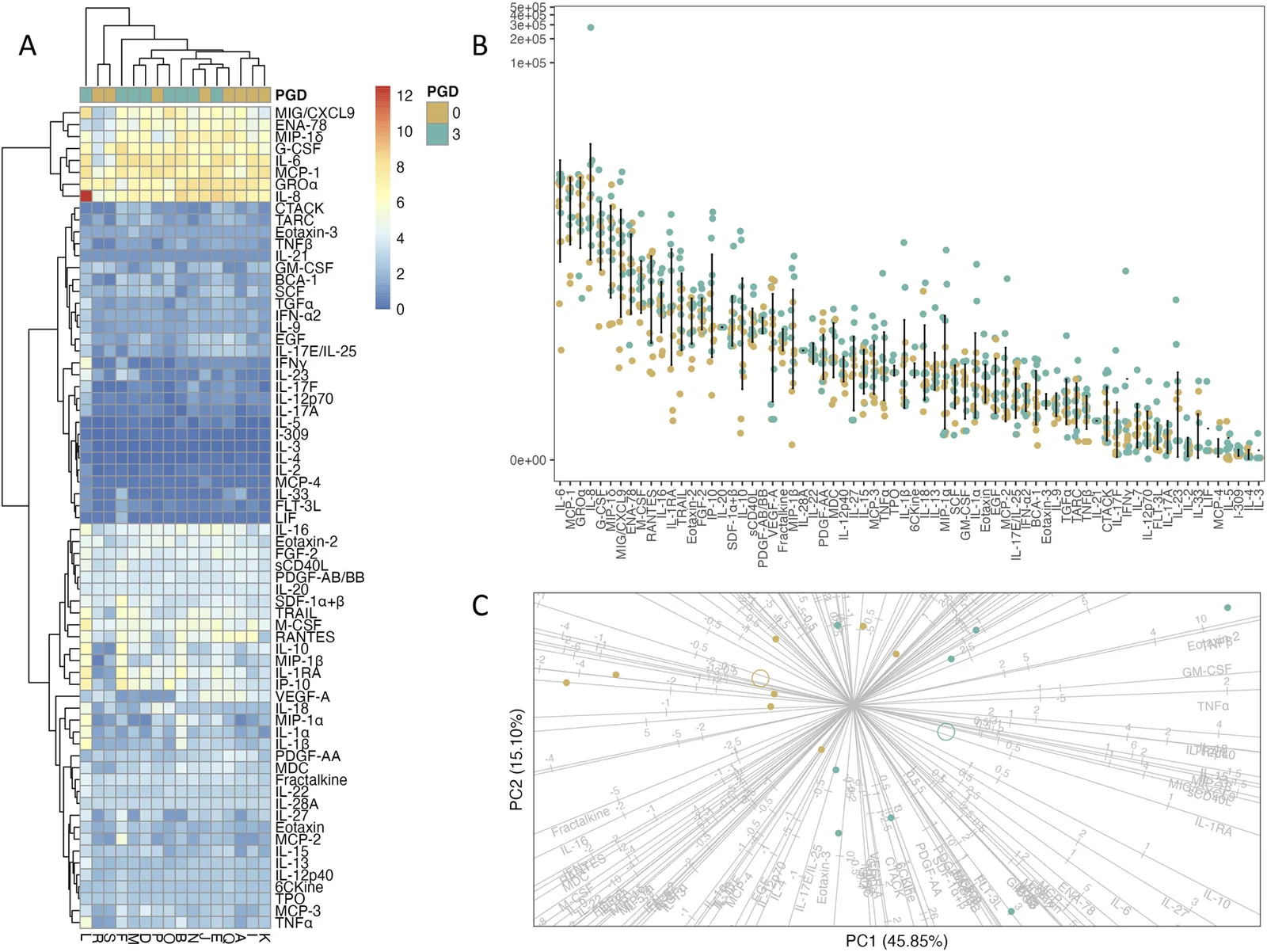
Differences in protein log-concentrations in BAL fluid by primary graft dysfunction (PGD) negative versus severe PGD 3. Differences in the levels of several proteins were detected between PGD–and PGD+ subjects. **(A)** Clustered heatmap for all subjects at T0, using complete-linkage clustering on cases and proteins, annotated by PGD grade. **(B)** Jitter plots, grouped by PGD grade. Proteins are in order of decreasing median concentration. **(C)** PCA biplot on all available protein concentrations. PGD group averages are indicated by open circles.

**TABLE 5:** Results of Kolmogorov–Smirnov tests of differences between undetected and severe primary graft dysfunction cases in protein concentrations in BAL fluid.

Protein	*n*_0_	*n*_3_	Statistic	p-value
IL-1RA	8	8	0.875	0.00249
Fractalkine BCA-1 Eotaxin	8 8 8	8 8 8	0.750 0.750 0.750	0.01010 0.01865 0.01865
IL-12p40	8	8	0.750	0.01865
IL-6 IP-10	8 8	180 8	0.750 0.750	0.01865 0.01865
M-CSF	8	8	0.750	0.01865

Analytes are listed in order of statistical evidence, up to unadjusted p < 0.05. See Supplementary Table 1 for details on the full set of analytes. Abbreviations; BCA-1, B-cell-attracting chemokine 1 (CXCL13); 6CKine, secondary lymphoid-tissue chemokine (CCL21); IL-22, interleukin-22; IL-16, interleukin-16; MCP-4, monocyte chemoattractant protein 4 (CCL13); IL-6, interleukin-6; MCP-1, monocyte chemoattractant protein 1 (CCL2); IL-15, interleukin-15; I-309, T-lymphocyte-secreted protein I-309 (CCL1); PDGF-AA, platelet-derived growth factor-AA; IL-12p40, interleukin-12 p40 subunit; IL-3, interleukin-3; TRAIL, TNF-related apoptosis-inducing ligand; PDGF-AB/BB, platelet-derived growth factor-AB/BB; MIP-1δ, macrophage inflammatory protein-1 delta (CCL15).

### Predictive modelling of PGD

The lack of strong associations between analyte concentrations and the PGD group using classical statistics led us to consider machine learning as an alternative approach. Keeping with classical statistics, machine learning models showed that BAL fluid is the specimen type with the greatest predictive value. Supplementary Tables 2, 3 in the Methodological Supplement compare model performance across each predictor set. Supplementary Table 2 presents the highest AUROC. To assess the sensitivity of these results to parameter tuning and moderate expectations for out-of-box performance, AUROCs in Supplementary Table 3 are averaged from all models using near-optimal parameter settings. Given this, we next built a reduced model using only BAL data. After excluding NN models for worst performance and difficulty of importance measurement, we used a custom cumulative importance measure and a formalized “elbow method” cutoff to select candidate biomarkers (I-309, Fractalkine, IL-12p40, IL-5, FGF-2, PDGF-AA, IL-1RA, M-CSF, IL-6, BCA-1, IP-10, and Eotaxin). Using regularized logistic regression analysis of these 12 analytes, four (I-309, IL-5, PDGF-AA, and Eotaxin) were eliminated (Figure 6A). Subjectively, we selected three analytes, IL-1Ra, Fractalkine, and BCA-1, to determine their potential as candidates for predicting severe PGD. Figure 6B shows the link function and its values taken at all cases, showing that the model accurately predicts PGD in every case. A PCA of the 16 cases used to optimize the BAL models on these analytes showed that they clearly separate PGD subgroups (Figure 6C). As we had exhausted the available cases in the nested CV process, used to obtain optimal models, we could not further validate this model. Therefore, we used classical diagnostics and randomized quantile residuals [37] to evaluate goodness of fit. These identified some influential outliers. The same clinical predictor pool was used consistently across all models that included clinical data, either alone or in combination with biological specimen data. To assess the importance of specific clinical factors, optimized AUROCs for logistic regression models on a single clinical predictor each are reported in the Methodological Supplement (Supplementary Figure S4). These analyses were performed to contextualize the relative contribution of clinical variables compared with multiplex cytokine measurements.

**FIGURE 6:**
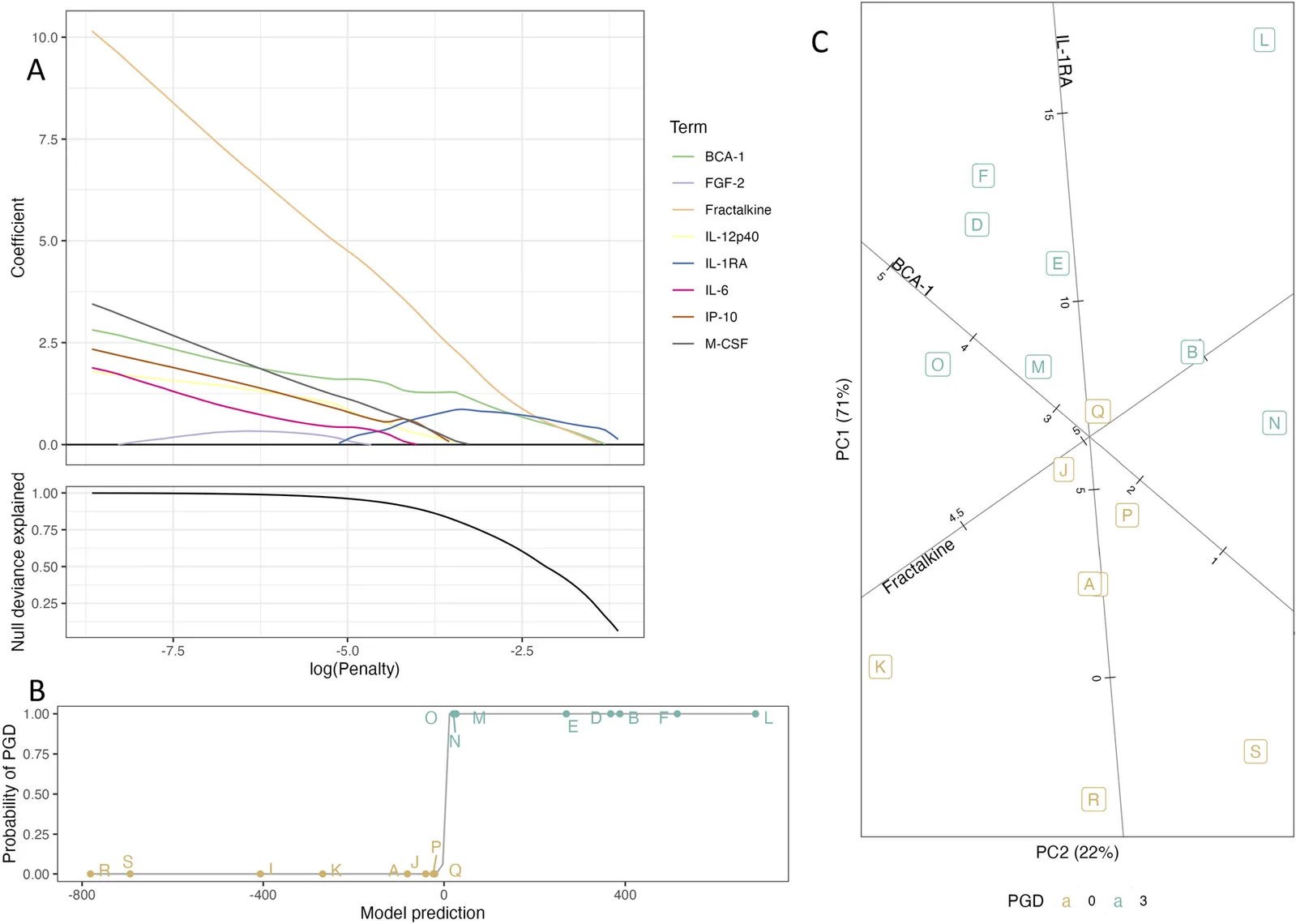
Selecting analytes for a candidate bronchoalveolar lavage protein panel. **(A)** Regularized regression coefficients and proportion of deviance explained in a LASSO on non-imputed multiplex assay data using eight analytes: BCA-1, FGF-2, Fractalkine, IL-12p40, IL-1Ra, IL-6, IP-10, and M-CSF. **(B)** PGD probability versus linear prediction for 16 cases, derived from a 3-analyte model (BCA-1, Fractalkine, IL-1Ra) informed by the LASSO. **(C)** PCA biplot of 16 cases used in BAL models on the 3 analytes included in the candidate panel. Abbreviations; BCA-1, B-cell-attracting chemokine 1 (CXCL13); FGF-2, fibroblast growth factor 2; Fractalkine, CX3C chemokine ligand 1 (CX3CL1); IL-12p40, interleukin-12 p40 subunit; IL-1Ra, interleukin-1 receptor antagonist; IL-6, interleukin-6; IP-10, interferon-γ-induced protein 10 (CXCL10); and M-CSF, macrophage colony-stimulating factor (CSF1), LASSO, least absolute shrinkage and selection operator.

### Robustness checks

To enhance the rigor of our approach we performed several robustness checks, as detailed in the methodological supplement.

## Discussion

Here we sought to identify donor and recipient inflammatory risk factors for the development of PGD to characterize its pathophysiology using a “hypothesis-free” approach. Our 71-analyte multiplex panel allowed us to examine previously reported markers of PGD while achieving unbiased discovery of new potential mechanistic/therapeutic targets and/or novel biomarkers. We sampled multiple biological sites from each recipient and obtained results using the same assay and data analysis methodology, allowing us to compare the utility of each sample site against each other without bias from batch variability. It is important to distinguish the two analytical aims of this study. First, we performed associative analyses across plasma, perfusate, and BAL to identify cytokines linked to PGD status or peri-transplant temporal changes. Second, we used predictive modeling to compare the relative utility of different specimen types for discrimination of severe PGD and to prioritize a reduced set of candidate BAL analytes. Thus, the longitudinal plasma analyses were intended primarily to define peri-transplant inflammatory patterns and persistence of candidate signals over time, whereas predictive modeling focused on early specimen types most relevant to acute PGD classification. Our analysis demonstrated the presence of previously undescribed factors associated with PGD in these different biological samples and determined that T0 BAL had the strongest PGD predictive value.

To our knowledge, no previous human studies have investigated cytokine levels in static donor lung preservation solution. In keeping with a recent heart transplant multiplex study of heart preservation solutions [38], we identified a large array of detectable factors in lung perfusate, as 66 of the 71 cytokines were detected. Of the 66 identified cytokines, only IL-17a was detectably different between PGD groups. Our plasma studies provide insight into cytokine level changes across the transplant process and determine whether signatures of cytokines are associated with severe PGD. Fifteen analytes were detectably different in PGD+ vs. PGD-plasma at individual time points. Amongst these were IL-6 and MCP-1, which have been previously reported to be elevated in the plasma of PGD patients [5, 8–11]. While 15 analytes were detectably changed, only two, BCA-1 and IL-22, were detectably elevated in PGD at all timepoints, using multivariate approaches. To our knowledge, these cytokines have not been reported as plasma PGD biomarkers thus far and may indicate that PGD patients have increased early B cell signaling [39, 40]. Finally, we utilized T0 BAL as a window into the local lung microenvironment. A large array of analytes was present in BAL. Of the 71 analytes assessed, we demonstrated that eight (IL-1RA, Fractalkine, BCA-1, Eotaxin, IL-12p40, IL-6, IP-10, and M-CSF) were detectably elevated in PGD.

Using classical statistics, we identified novel analytes that have not been previously described or linked mechanistically in PGD. A secondary goal of our study was to explore whether our multiplex approach could optimize the identification of biomarkers that predict severe PGD. While most biomarker discovery studies use only statistical association tests or univariable prediction models to identify candidates, a growing number use multivariable models, often internally cross-validated [41]. For example, Wolf *et al* [42] and Berra *et al* [43] used proteins measured in BAL by mass spectrometry or Western blot, in concert with FEV, to build machine learning models that successfully stratified the risk of CLAD development. To our knowledge, no multivariable studies have utilized patient-matched biospecimens from different sites to test multiplex data and sample types. Here we show that our multivariate predictive modeling comparisons indicated the optimum sample to analyze for its capacity to predict PGD onset, in our sample types, was BAL collected 2hrs post-transplant (T0). Using this exploratory reduction strategy, we prioritized IL-1Ra, CXCL13/BCA-1, and Fractalkine as candidate BAL analytes associated with severe PGD. Using a LASSO analysis to assess the predictive power of analytes in T0 BAL specimen we identified three cytokines, IL-1Ra, BCA-1, and Fractalkine, as strong candidates that accurately predict PGD while minimizing model deviance. Except for IL-1Ra, these cytokines have not been previously correlated to PGD development. Interestingly, BCA-1 levels were also detectably elevated in plasma, both pre- and post-transplantation. Given the mechanism of action of BCA-1 [44, 45], our findings are interesting in light of recent studies implicating B cells in lung transplant IRI [46–48]. While not investigated in lung IRI or lung inflammation, Fractalkine has been shown to play important roles in the context of cardiac IRI [49–51] and inflammation [52, 53]. Taken together, our findings support the need for future functional studies investigating the roles of IL-1Ra, BCA-1, and Fractalkine to fully evaluate the significance of these mediators in PGD.

Recent studies have utilized cytokine rapid testing to identify and stratify patients at risk of hospitalization upon presenting with respiratory distress [54]. Given the early post-transplant signal of IL-1Ra, BCA-1, and Fractalkine, observed in BAL, these findings raise the possibility that a focused cytokine panel could ultimately contribute to early PGD risk stratification. Our predictive modeling passed multiple data robustness checks. However, the present study is not sufficient to establish clinical utility, and any point-of-care application will require external validation, calibration testing, and assessment in broader cohorts that include intermediate and fluctuating PGD phenotypes. As such, these markers should not yet be considered a validated predictive panel but rather a biologically plausible and clinically testable candidate signature for follow-up studies.

Previous multiplex cytokine studies in cardiac transplantation showed similar large-scale cytokine changes at different time points post-transplant and note, as we have, new target cytokines [38]. A major difference in our study, beyond its lung focus, was the inclusion of patients with different early graft outcomes. By incorporating PGD+ and PGD-patients, we were able to not only map cytokine changes across sample type and time but further identified novel analytes that could have mechanistic and predictive value for the discrimination of PGD. To promote dichotomization in our machine learning models, we included patients that were either PGD 0 or 3 at all timepoints (24, 48, and 72 h). We acknowledge the accepted PGD construct is to collapse the 0–72 h grades 1–3 into PGD3 at 48 or 72h vs. no PGD3 at 48 or 72 h^1^. However, we believe our approach optimized its discovery and predictive ability given the relatively small sample size. Nevertheless, future studies are needed to validate the analytes we discovered using a larger sample cohort and accepted PGD diagnostic construct.

This study has several important limitations. Most notably, the cohort size was small relative to the dimensionality of the multiplex and modeling analyses, increasing the risk of model instability and overfitting. Accordingly, the very high predictive performance observed in some combined-feature models, including near-perfect AUROC values, should not be interpreted as evidence of a clinically ready classifier but rather as support for this framework as an exploratory approach to prioritize candidate biomarkers and informative specimen types. We focused our studies on the extremes of PGD, with our two groups split between PGD0 and PGD3 at all timepoints. We recognize that this approach does not capture the full clinical spectrum of PGD nor establish how identified markers would perform in intermediate phenotypes. Evaluation of PGD grades 1–2 and temporally dynamic trajectories will require substantially larger cohorts and will be the focus of future validation studies.

Baseline differences between PGD groups may also have influenced the observed biological signals. Although available clinical predictors were evaluated individually and in combination with biological data, and no single measured clinical variable performed comparably to the BAL cytokine signal, residual confounding cannot be excluded. In addition, several relevant donor and preservation variables, including ischemic time, donor PaO2/FiO2, ventilator settings, and flush volume, were unavailable in the de-identified tissue bank dataset, limiting our ability to assess their contribution to group differences.

Taken together, these considerations emphasize that the identified BAL analytes, including IL-1Ra, CXCL13/BCA-1, and Fractalkine, should be regarded as hypothesis-generating candidates. Validation in larger, independent, prospectively collected cohorts with more complete donor and perioperative annotation will be required.

In conclusion, we used matched perfusate, plasma, and BAL biospecimens to define inflammatory signatures associated with severe PGD after LTx. Across specimen types, early BAL demonstrated the clearest relationship with PGD status and identified BCA-1, Fractalkine, and IL-1Ra as candidate analytes for further study, supporting their potential relevance to PGD pathophysiology and the need for future mechanistic investigation. These findings should be interpreted cautiously given the limited cohort size, incomplete availability of certain donor and preservation variables, and the exploratory nature of the analyses. Nonetheless, this study provides a framework for integrating multiplex profiling with multivariable machine learning approaches to nominate biologically relevant, testable biomarkers and mechanistic targets for future translational PGD studies.

## Data Availability

The original contributions presented in the study are included in the article/Supplementary Material, further inquiries can be directed to the corresponding author.

## References

[1] Christie JD Bellamy S Ware LB Lederer D Hadjiliadis D Lee J Construct validity of the definition of primary graft dysfunction after lung transplantation. The J Heart Lung Transplant (2010) 29(11):1231–9. 10.1016/j.healun.2010.05.013 20655249 PMC2963709

[2] Huang HJ Yusen RD Meyers BF Walter MJ Mohanakumar T Patterson GA Late primary graft dysfunction after lung transplantation and bronchiolitis obliterans syndrome. Am J Transplant Official J Am Soc Transplant Am Soc Transpl Surgeons (2008) 8(11):2454–62. 10.1111/j.1600-6143.2008.02389.x 18785961 PMC2678949

[3] Daud SA Yusen RD Meyers BF Chakinala MM Walter MJ Aloush AA Impact of immediate primary lung allograft dysfunction on bronchiolitis obliterans syndrome. Am J Respir Crit Care Med (2007) 175(5):507–13. 10.1164/rccm.200608-1079OC 17158279

[4] Kreisel D Krupnick AS Puri V Guthrie TJ Trulock EP Meyers BF Short- and long-term outcomes of 1000 adult lung transplant recipients at a single center. The J Thorac Cardiovasc Surg (2011) 141(1):215–22. 10.1016/j.jtcvs.2010.09.009 21093882

[5] Moreno I Vicente R Ramos F Vicente JL Barberá M . Determination of Interleukin-6 in lung transplantation: association with primary graft dysfunction. Transplant Proc (2007) 39(7):2425–6. 10.1016/j.transproceed.2007.07.056 17889209

[6] Chacon-Alberty L Kanchi RS Ye S Hochman-Mendez C Daoud D Coarfa C Plasma protein biomarkers for primary graft dysfunction after lung transplantation: a single-center cohort analysis. Scientific Rep (2022) 12(1):16137. 10.1038/s41598-022-20085-y PMC9515157 36167867

[7] Mathur A Baz M Staples ED Bonnell M Speckman JM Hess PJ Cytokine profile after lung transplantation: cor-relation with allograft injury. The Ann Thorac Surg (2006) 81(5):1844–50. 10.1016/j.athoracsur.2005.11.053 16631683

[8] Hoffman SA Wang L Shah CV Ahya V Surname G Pochettino A Plasma cytokines and chemokines in primary graft dysfunction post-lung transplantation. Am J Transplant Official J Am Soc Transplant Am Soc Transpl Surgeons (2009) 9(2):389–96. 10.1111/j.1600-6143.2008.02497.x PMC2821938 19120076

[9] Bharat A Kuo E Steward N Aloush A Hachem R Trulock EP Immunological link between primary graft dysfunction and chronic lung allograft rejection. The Ann Thorac Surg (2008) 86(1):189–97. 10.1016/j.athoracsur.2008.03.073 18573422 PMC2790810

[10] Shah RJ Bellamy SL Localio AR Wickersham N Diamond JM Weinacker A A panel of lung injury biomarkers enhances the definition of primary graft dysfunction (PGD) after lung transplantation. The J Heart Lung Transplant (2012) 31(9):942–9. 10.1016/j.healun.2012.05.001 22694851 PMC3418416

[11] Bonneau S Landry C Bégin S Adam D Villeneuve L Clavet-Lanthier MÉ Correlation between neutrophil extracellular traps (NETs) expression and primary graft dysfunction following human lung transplantation. Cells (2022) 11(21):3420. 10.3390/cells11213420 PMC9656095 36359815

[12] Allen JG Lee MT Weiss ES Arnaoutakis GJ Shah AS Detrick B . Preoperative recipient cy-tokine levels are associated with early lung allograft dysfunction. The Ann Thorac Surg (2012) 93(6):1843–9. 10.1016/j.athoracsur.2012.02.041 22503850

[13] Christie JD Robinson N Ware LB Plotnick M De Andrade J Lama V Association of protein C and type 1 plasminogen activator inhibitor with primary graft dysfunction. Am J Respir Crit Care Med (2007) 175(1):69–74. 10.1164/rccm.200606-827OC 17023732 PMC1899260

[14] Frick AE Verleden SE Ordies S Sacreas A Vos R Verleden GM Early protein expression profile in bronchoalveolar lavage fluid and clinical outcomes in primary graft dysfunction after lung transplantation. Eur J Cardio-Thoracic Surg (2020) 58(2):379–88. 10.1093/ejcts/ezaa043 32267918

[15] Verleden SE Martens A Ordies S Neyrinck AP Van Raemdonck DE Verleden GM Immediate post-operative broncho-alveolar lavage IL -6 and IL -8 are associated with early outcomes after lung transplantation. Clin Transplant (2018) 32(4):e13219. 10.1111/ctr.13219 29405435

[16] DerHovanessian A Weigt SS Palchevskiy V Shino MY Sayah DM Gregson AL The role of TGF-*β* in the association between primary graft dysfunction and bronchiolitis obliterans syndrome. Am J Transplan- Tation: Official J Am Soc Transplant Am Soc Transpl Surgeons. (2016) 16(2):640–9. 10.1111/ajt.13475 26461171 PMC4946573

[17] Machuca TN Cypel M Yeung JC Bonato R Zamel R Chen M Protein expression profiling predicts graft Per-formance in clinical *Ex Vivo* lung perfusion. Ann Surg (2015) 261(3):591–7. 10.1097/SLA.0000000000000974 25371129

[18] Ghaidan H Stenlo M Niroomand A Mittendorfer M Hirdman G Gvazava N Reduction of primary graft dysfunction using cytokine adsorption during organ preservation and after lung transplantation. Nat Commun (2022) 13(1):4173. 10.1038/s41467-022-31811-5 PMC9325745 35882835

[19] Snell GI Yusen RD Weill D Strueber M Garrity E Reed A Report of the ISHLT working group on primary lung graft dysfunction, part I: definition and grading—A 2016 consensus group statement of the international society for heart and lung transplantation. The J Heart Lung Transplant (2017) 36(10):1097–103. 10.1016/j.healun.2017.07.021 28942784

[20] Martinu T Koutsokera A Benden C Cantu E Chambers D Cypel M International society for heart and lung transplantation consensus statement for the standardization of bronchoalveolar lavage in lung transplantation. The J Heart Lung Transplant The Official Publ Int Soc Heart Transplant (2020) 39(11):1171–90. 10.1016/j.healun.2020.07.006 PMC7361106 32773322

[21] R Core Team. R: A Language and Environment for Statistical Computing (2021).

[22] Wickham H Averick M Bryan J Chang W McGowan L François R Welcome to the tidyverse. J Open Source Softw (2019) 4(43):1686. 10.21105/joss.01686

[23] Kuhn M Wickham H . Tidymodels: A Collection of Packages for Modeling and Machine Learning Using Tidyverse Principles (2020).

[24] Wickham H Bryan J . Readxl: Read Excel Files (2023).

[25] Tierney N Cook D McBain M Fay C O’Hara-Wild, Hester J Naniar: Data Structures, Summaries, and Visualisations for Missing Data (2023).

[26] Tierney N Hughes S Averick M Lee S Wang E Crane N Visdat: Preliminary Visualisation of Data (2023).

[27] Kolde R . Pheatmap: Pretty Heatmaps (2019).

[28] Wei T Simko V Levy M . Corrplot: Visualization of a Correlation Matrix (2021).

[29] Brunson JC Paul E . Ordr: A ’Tidyverse’ Extension for Ordinations and Biplots (2022).

[30] Dahl DB Scott D Roosen C . Xtable: Export Tables to Latex or HTML (2019).

[31] Greenwell BM Boehmke B . Vip: Variable Importance Plots (2023).

[32] Pedersen TL . Tidygraph: A Tidy API for Graph Manipulation (2024).

[33] Pedersen TL . Ggraph: An Implementation of Grammar of Graphics for Graphs and Networks (2024).

[34] Galili T Benjamini Y Simpson G . Dendextend: Extending ’Dendrogram’ Functionality in R (2023).

[35] Kaplan J Schlegel B . Fastdummies: Fast Creation of Dummy (Binary) Columns and Rows from Categorical Variables (2023).

[36] Baringhaus L Franz C . On a new multivariate two-sample test. J Multivariate Anal (2004) 88(1):190–206. 10.1016/S0047-259X(03)00079-4

[37] Dunn PK Smyth GK . Randomized quantile residuals. J Comput Graphical Stat (1996) 5(3):236–44. 10.1080/10618600.1996.10474708

[38] Ledwoch N Wiegmann B Chichelnitskiy E Wandrer F Kühne JF Beushausen K Identification of distinct secretory patterns and their regulatory networks of ischemia *versus* reperfusion phases in clinical heart transplantation. Cytokine (2022) 149:155744. 10.1016/j.cyto.2021.155744 34649160

[39] Mao Y Wang M Zhou Q Jin J Peng W Wu J CXCL10 and CXCL13 expression were highly Up-regulated in peripheral blood mononuclear cells in acute rejection and poor response to anti-rejection therapy. J Clin Immunol (2011) 31(3):414–8. 10.1007/s10875-010-9500-8 21191639

[40] Arshad T Mansur F Palek R Manzoor S Liska V . A double edged sword role of Interleukin-22 in wound healing and tissue regeneration. Front Immunol (2020) 11:2148. 10.3389/fimmu.2020.02148 33042126 PMC7527413

[41] Halloran K Mackova M Parkes MD Hirji A Weinkauf J Timofte IL The molecular features of chronic lung allograft dysfunction in lung transplant airway mucosa. The J Heart Lung Transplant (2022) 41(12):1689–99. 10.1016/j.healun.2022.08.014 36163162

[42] Wolf T Oumeraci T Gottlieb J Pich A Brors B Eils R Proteomic bronchiolitis Obliterans syndrome risk monitoring in lung transplant recipients. Transplantation (2011) 92(4):477–85. 10.1097/TP.0b013e318224c109 21716170

[43] Berra G Farkona S Mohammed-Ali Z Kotlyar M Levy L Clotet-Freixas S Association between renin-angiotensin system and chronic lung allograft dysfunction. Eur Respir J (2021) 58:2002975. 10.1183/13993003.02975-2020 33863738

[44] Legler DF Loetscher M Roos RS Clark-Lewis I Baggiolini M Moser B . B cell-attracting chemokine 1, a human CXC chemokine expressed in lymphoid tissues, selectively attracts B lymphocytes *via* BLR1/CXCR5. The J Exp Med (1998) 187(4):655–60. 10.1084/jem.187.4.655 9463416 PMC2212150

[45] Jenh CH Cox MA Hipkin W Lu T Pugliese-Sivo C Gonsiorek W Human B cell-attracting chemokine 1 (BCA-1; CXCL13) is an agonist for the human CXCR3 receptor. Cytokine (2001) 15(3):113–21. 10.1006/cyto.2001.0923 11554781

[46] Farahnak K Bai YZ Yokoyama Y Morkan DB Liu Z Amrute JM B cells mediate lung ischemia/reperfusion injury by recruiting classical monocytes *via* synergistic B cell receptor/TLR4 signaling. The J Clin Invest (2024) 134(6):e170118. 10.1172/JCI170118 PMC10940088 38488011

[47] Li W Gauthier JM Higashikubo R Hsiao HM Tanaka S Vuong L Bronchus-associated lymphoid tissue-resident Foxp3+ T lymphocytes prevent antibody-mediated lung rejection. The J Clin Invest (2019) 129(2):556–68. 10.1172/JCI122083 30561386 PMC6355223

[48] Tanaka S Gauthier JM Fuchs A Li W Tong AY Harrison MS IL-22 is required for the induction of bronchus-associated lymphoid tissue in tolerant lung allografts. Am J Transplant Official J Am Soc Transplant Am Soc Transpl Surgeons (2020) 20(5):1251–61. 10.1111/ajt.15701 31721409 PMC7183893

[49] Grosse GM Tryc AB Dirks M Schuppner R Pflugrad H Lichtinghagen R The temporal dynamics of plasma fractalkine levels in ischemic stroke: association with clinical severity and outcome. J Neuroinflammation (2014) 11:74. 10.1186/1742-2094-11-74 24722201 PMC4022085

[50] Kanzawa T Tokita D Saiga K Yamakawa T Ishigooka H Fukuda H Role of Fractalkine-CX3CR1 axis in acute rejection of mouse heart allografts subjected to ischemia reperfusion injury. Transpl Int Official J Eur Soc Organ Transplant (2022) 35:10157. 10.3389/ti.2022.10157 PMC8842273 35185378

[51] Loh SX Ekinci Y Spray L Jeyalan V Olin T Richardson G Fractalkine signalling (CX3CL1/CX3CR1 axis) as an emerging target in coronary artery disease. J Clin Med (2023) 12(14):4821. 10.3390/jcm12144821 PMC10381654 37510939

[52] Arend WP . The balance between IL-1 and IL-1Ra in disease. Cytokine and Growth Factor Rev (2002) 13(4-5):323–40. 10.1016/s1359-6101(02)00020-5 12220547

[53] Griffith JW Faustino LD Cottrell VI Nepal K Hariri LP Chiu RSY Regulatory T cell-derived IL-1Ra suppresses the innate response to respiratory viral infection. Nat Immunol (2023) 24(12):2091–107. 10.1038/s41590-023-01655-2 PMC11887468 37945820

[54] Husain S Sage AT Del Sorbo L Cypel M Martinu T Juvet SC A biomarker assay to risk-stratify patients with symp-toms of respiratory tract infection. The Eur Respir J (2022) 60(6):2200459. 10.1183/13993003.00459-2022 PMC9753477 36104292

