# Supplementary material for: Multi-site cytokine levels are predictive of primary graft dysfunction following lung transplantation
Source: Transpl Int. 2026 Sep 14;39:15874. doi: 10.3389/ti.2026.15874 (PMC13616802; doi:10.3389/ti.2026.15874)
Supplement: Supplementary file 1 [file DataSheet1.pdf]

Methodological Supplement for “Predicting Primary Graft Dysfunction in Lung Transplantation: Machine Learning–Guided Biomarker Discovery”

2026 February 22

Abstract

This supplemental text explains the methodology of the study in more detail.

Preprocessing

Donor organ data

Donor age, gender, race, and smoking and alcohol history were collected. Donor smoking and alcohol history were recoded to yes/no responses. All organs were procured after brain death. Storage temperature was coded as standard or normothermic.

Assay data

Plasma samples were taken at 6 time points, coded as “pre” (specimens taken within 12hr before transplantation), “2hr” (specimens taken within 2 hours following transplantation, designated in the text as “T0”), “24hr”, “72hr”, “1wk”, or “2wk” (specimens taken at 14 or 15 days). We used only provided average values in our analysis.

Samples were collected and analyzed from 17 cases (9 PGD–, 8 PGD+). However, multiplex data from perfusate, BAL fluid, and plasma at 2wk were not obtained for an additional PGD– case, from plasma at 2wk for an additional PGD– case, and from perfusate for an additional PGD+ case. While only 13 cases had complete data, analyses using data from only some sites included 15 cases or more. Table M1 reports completeness rates for all proteins.

Table M1: Number and percent of cases for which concentration values were calculatable for each protein.

Medium	Time	Protein	PGD–	PGD+
perfusate	pre	6CKine	6/8 (75.0%)	7/7 (100.0%)
perfusate	pre	EGF	3/8 (37.5%)	3/7 (42.9%)
perfusate	pre	ENA-78	7/8 (87.5%)	7/7 (100.0%)
perfusate	pre	Eotaxin-3	3/8 (37.5%)	2/7 (28.6%)
perfusate	pre	Fractalkine	7/8 (87.5%)	7/7 (100.0%)
perfusate	pre	GM-CSF	6/8 (75.0%)	6/7 (85.7%)
perfusate	pre	IFN-α2	7/8 (87.5%)	7/7 (100.0%)
perfusate	pre	IFNγ	1/8 (12.5%)	0/7 (0.0%)
perfusate	pre	IL-12p70	5/8 (62.5%)	6/7 (85.7%)
perfusate	pre	IL-17A	6/8 (75.0%)	4/7 (57.1%)
perfusate	pre	IL-17E/IL-25	0/8 (0.0%)	1/7 (14.3%)
perfusate	pre	IL-17F	5/8 (62.5%)	4/7 (57.1%)
perfusate	pre	IL-1β	7/8 (87.5%)	7/7 (100.0%)
perfusate	pre	IL-2	5/8 (62.5%)	3/7 (42.9%)

Medium	Time	Protein	PGD–	PGD+
perfusate	pre	IL-20	0/8 (0.0%)	0/7 (0.0%)
perfusate	pre	IL-21	0/8 (0.0%)	0/7 (0.0%)
perfusate	pre	IL-22	1/8 (12.5%)	0/7 (0.0%)
perfusate	pre	IL-23	5/8 (62.5%)	3/7 (42.9%)
perfusate	pre	IL-27	7/8 (87.5%)	5/7 (71.4%)
perfusate	pre	IL-28A	2/8 (25.0%)	3/7 (42.9%)
perfusate	pre	IL-3	0/8 (0.0%)	0/7 (0.0%)
perfusate	pre	IL-33	7/8 (87.5%)	7/7 (100.0%)
perfusate	pre	IL-4	1/8 (12.5%)	1/7 (14.3%)
perfusate	pre	IL-7	1/8 (12.5%)	4/7 (57.1%)
perfusate	pre	IL-9	7/8 (87.5%)	7/7 (100.0%)
perfusate	pre	LIF	0/8 (0.0%)	0/7 (0.0%)
perfusate	pre	MCP-2	4/8 (50.0%)	3/7 (42.9%)
perfusate	pre	MCP-4	2/8 (25.0%)	0/7 (0.0%)
perfusate	pre	MIP-1α	1/8 (12.5%)	1/7 (14.3%)
perfusate	pre	TNFβ	2/8 (25.0%)	2/7 (28.6%)
perfusate	pre	TPO	6/8 (75.0%)	6/7 (85.7%)
perfusate	pre	TSLP	0/8 (0.0%)	0/7 (0.0%)
perfusate	pre	VEGF-A	1/8 (12.5%)	0/7 (0.0%)
plasma	pre	6CKine	8/9 (88.9%)	6/8 (75.0%)
plasma	pre	EGF	7/9 (77.8%)	5/8 (62.5%)
plasma	pre	ENA-78	8/9 (88.9%)	8/8 (100.0%)
plasma	pre	Eotaxin-3	2/9 (22.2%)	2/8 (25.0%)
plasma	pre	FLT-3L	9/9 (100.0%)	7/8 (87.5%)
plasma	pre	GM-CSF	8/9 (88.9%)	7/8 (87.5%)
plasma	pre	GROα	9/9 (100.0%)	6/8 (75.0%)
plasma	pre	IFN-α2	6/9 (66.7%)	6/8 (75.0%)
plasma	pre	IFNγ	7/9 (77.8%)	8/8 (100.0%)
plasma	pre	IL-10	4/9 (44.4%)	6/8 (75.0%)
plasma	pre	IL-12p40	5/9 (55.6%)	6/8 (75.0%)
plasma	pre	IL-12p70	7/9 (77.8%)	7/8 (87.5%)
plasma	pre	IL-13	5/9 (55.6%)	6/8 (75.0%)
plasma	pre	IL-15	3/9 (33.3%)	6/8 (75.0%)
plasma	pre	IL-16	7/9 (77.8%)	5/8 (62.5%)
plasma	pre	IL-17A	7/9 (77.8%)	4/8 (50.0%)
plasma	pre	IL-17F	9/9 (100.0%)	7/8 (87.5%)
plasma	pre	IL-2	2/9 (22.2%)	3/8 (37.5%)
plasma	pre	IL-20	2/9 (22.2%)	0/8 (0.0%)
plasma	pre	IL-21	3/9 (33.3%)	1/8 (12.5%)
plasma	pre	IL-22	3/9 (33.3%)	6/8 (75.0%)
plasma	pre	IL-23	3/9 (33.3%)	2/8 (25.0%)
plasma	pre	IL-28A	1/9 (11.1%)	1/8 (12.5%)
plasma	pre	IL-3	4/9 (44.4%)	3/8 (37.5%)
plasma	pre	IL-33	3/9 (33.3%)	2/8 (25.0%)
plasma	pre	IL-7	8/9 (88.9%)	7/8 (87.5%)
plasma	pre	IL-9	3/9 (33.3%)	3/8 (37.5%)
plasma	pre	LIF	5/9 (55.6%)	5/8 (62.5%)
plasma	pre	M-CSF	9/9 (100.0%)	6/8 (75.0%)
plasma	pre	MCP-4	9/9 (100.0%)	6/8 (75.0%)
plasma	pre	MIP-1α	7/9 (77.8%)	5/8 (62.5%)
plasma	pre	SCF	4/9 (44.4%)	5/8 (62.5%)
plasma	pre	TGFα	8/9 (88.9%)	5/8 (62.5%)

Medium	Time	Protein	PGD–	PGD+
plasma	pre	TNFβ	2/9 (22.2%)	3/8 (37.5%)
plasma	pre	TPO	6/9 (66.7%)	6/8 (75.0%)
plasma	pre	TSLP	3/9 (33.3%)	3/8 (37.5%)
plasma	2hr	EGF	5/9 (55.6%)	7/8 (87.5%)
plasma	2hr	Eotaxin-2	9/9 (100.0%)	7/8 (87.5%)
plasma	2hr	Eotaxin-3	2/9 (22.2%)	0/8 (0.0%)
plasma	2hr	FLT-3L	8/9 (88.9%)	8/8 (100.0%)
plasma	2hr	IFN-α2	8/9 (88.9%)	7/8 (87.5%)
plasma	2hr	IFNγ	8/9 (88.9%)	8/8 (100.0%)
plasma	2hr	IL-12p40	6/9 (66.7%)	5/8 (62.5%)
plasma	2hr	IL-13	6/9 (66.7%)	8/8 (100.0%)
plasma	2hr	IL-16	6/9 (66.7%)	7/8 (87.5%)
plasma	2hr	IL-17A	8/9 (88.9%)	8/8 (100.0%)
plasma	2hr	IL-17F	8/9 (88.9%)	8/8 (100.0%)
plasma	2hr	IL-2	6/9 (66.7%)	8/8 (100.0%)
plasma	2hr	IL-20	0/9 (0.0%)	0/8 (0.0%)
plasma	2hr	IL-21	1/9 (11.1%)	1/8 (12.5%)
plasma	2hr	IL-22	7/9 (77.8%)	8/8 (100.0%)
plasma	2hr	IL-23	1/9 (11.1%)	1/8 (12.5%)
plasma	2hr	IL-28A	1/9 (11.1%)	0/8 (0.0%)
plasma	2hr	IL-3	5/9 (55.6%)	3/8 (37.5%)
plasma	2hr	IL-33	1/9 (11.1%)	1/8 (12.5%)
plasma	2hr	IL-7	7/9 (77.8%)	8/8 (100.0%)
plasma	2hr	IL-9	2/9 (22.2%)	2/8 (25.0%)
plasma	2hr	LIF	6/9 (66.7%)	5/8 (62.5%)
plasma	2hr	MCP-4	6/9 (66.7%)	2/8 (25.0%)
plasma	2hr	SCF	5/9 (55.6%)	3/8 (37.5%)
plasma	2hr	TGFα	8/9 (88.9%)	8/8 (100.0%)
plasma	2hr	TNFβ	3/9 (33.3%)	2/8 (25.0%)
plasma	2hr	TPO	4/9 (44.4%)	2/8 (25.0%)
plasma	2hr	TSLP	1/9 (11.1%)	1/8 (12.5%)
plasma	2hr	VEGF-A	9/9 (100.0%)	7/8 (87.5%)
plasma	24hr	6CKine	8/9 (88.9%)	8/8 (100.0%)
plasma	24hr	EGF	6/9 (66.7%)	6/8 (75.0%)
plasma	24hr	ENA-78	7/9 (77.8%)	8/8 (100.0%)
plasma	24hr	Eotaxin-3	0/9 (0.0%)	0/8 (0.0%)
plasma	24hr	FLT-3L	8/9 (88.9%)	7/8 (87.5%)
plasma	24hr	GM-CSF	8/9 (88.9%)	8/8 (100.0%)
plasma	24hr	GROα	6/9 (66.7%)	8/8 (100.0%)
plasma	24hr	IFN-α2	6/9 (66.7%)	5/8 (62.5%)
plasma	24hr	IL-12p40	4/9 (44.4%)	2/8 (25.0%)
plasma	24hr	IL-12p70	8/9 (88.9%)	7/8 (87.5%)
plasma	24hr	IL-13	6/9 (66.7%)	6/8 (75.0%)
plasma	24hr	IL-16	8/9 (88.9%)	7/8 (87.5%)
plasma	24hr	IL-17A	6/9 (66.7%)	6/8 (75.0%)
plasma	24hr	IL-17E/IL-25	9/9 (100.0%)	7/8 (87.5%)
plasma	24hr	IL-17F	6/9 (66.7%)	3/8 (37.5%)
plasma	24hr	IL-20	0/9 (0.0%)	0/8 (0.0%)
plasma	24hr	IL-21	1/9 (11.1%)	0/8 (0.0%)
plasma	24hr	IL-22	6/9 (66.7%)	8/8 (100.0%)
plasma	24hr	IL-23	1/9 (11.1%)	1/8 (12.5%)
plasma	24hr	IL-28A	1/9 (11.1%)	0/8 (0.0%)

Medium	Time	Protein	PGD–	PGD+
plasma	24hr	IL-3	4/9 (44.4%)	3/8 (37.5%)
plasma	24hr	IL-33	1/9 (11.1%)	1/8 (12.5%)
plasma	24hr	IL-7	8/9 (88.9%)	8/8 (100.0%)
plasma	24hr	IL-9	2/9 (22.2%)	2/8 (25.0%)
plasma	24hr	LIF	3/9 (33.3%)	4/8 (50.0%)
plasma	24hr	MCP-4	4/9 (44.4%)	2/8 (25.0%)
plasma	24hr	MIP-1α	7/9 (77.8%)	7/8 (87.5%)
plasma	24hr	SCF	3/9 (33.3%)	2/8 (25.0%)
plasma	24hr	TGFα	7/9 (77.8%)	7/8 (87.5%)
plasma	24hr	TNFβ	2/9 (22.2%)	0/8 (0.0%)
plasma	24hr	TPO	5/9 (55.6%)	3/8 (37.5%)
plasma	24hr	TRAIL	9/9 (100.0%)	7/8 (87.5%)
plasma	24hr	TSLP	1/9 (11.1%)	1/8 (12.5%)
plasma	72hr	6CKine	9/9 (100.0%)	7/8 (87.5%)
plasma	72hr	EGF	7/9 (77.8%)	5/8 (62.5%)
plasma	72hr	ENA-78	9/9 (100.0%)	7/8 (87.5%)
plasma	72hr	Eotaxin-3	2/9 (22.2%)	1/8 (12.5%)
plasma	72hr	FLT-3L	9/9 (100.0%)	6/8 (75.0%)
plasma	72hr	GROα	6/9 (66.7%)	6/8 (75.0%)
plasma	72hr	IFN-α2	7/9 (77.8%)	5/8 (62.5%)
plasma	72hr	IFNγ	8/9 (88.9%)	8/8 (100.0%)
plasma	72hr	IL-12p40	5/9 (55.6%)	2/8 (25.0%)
plasma	72hr	IL-12p70	9/9 (100.0%)	6/8 (75.0%)
plasma	72hr	IL-13	6/9 (66.7%)	6/8 (75.0%)
plasma	72hr	IL-16	4/9 (44.4%)	6/8 (75.0%)
plasma	72hr	IL-17A	7/9 (77.8%)	6/8 (75.0%)
plasma	72hr	IL-17E/IL-25	9/9 (100.0%)	7/8 (87.5%)
plasma	72hr	IL-17F	8/9 (88.9%)	6/8 (75.0%)
plasma	72hr	IL-2	7/9 (77.8%)	7/8 (87.5%)
plasma	72hr	IL-20	0/9 (0.0%)	0/8 (0.0%)
plasma	72hr	IL-21	1/9 (11.1%)	1/8 (12.5%)
plasma	72hr	IL-22	6/9 (66.7%)	6/8 (75.0%)
plasma	72hr	IL-23	2/9 (22.2%)	3/8 (37.5%)
plasma	72hr	IL-28A	1/9 (11.1%)	0/8 (0.0%)
plasma	72hr	IL-3	7/9 (77.8%)	2/8 (25.0%)
plasma	72hr	IL-33	1/9 (11.1%)	2/8 (25.0%)
plasma	72hr	IL-5	8/9 (88.9%)	6/8 (75.0%)
plasma	72hr	IL-7	8/9 (88.9%)	7/8 (87.5%)
plasma	72hr	IL-9	6/9 (66.7%)	2/8 (25.0%)
plasma	72hr	LIF	7/9 (77.8%)	7/8 (87.5%)
plasma	72hr	MCP-2	9/9 (100.0%)	7/8 (87.5%)
plasma	72hr	MCP-4	5/9 (55.6%)	2/8 (25.0%)
plasma	72hr	MIP-1α	8/9 (88.9%)	7/8 (87.5%)
plasma	72hr	SCF	3/9 (33.3%)	2/8 (25.0%)
plasma	72hr	TNFβ	3/9 (33.3%)	0/8 (0.0%)
plasma	72hr	TPO	5/9 (55.6%)	6/8 (75.0%)
plasma	72hr	TSLP	1/9 (11.1%)	3/8 (37.5%)
plasma	1wk	EGF	6/9 (66.7%)	5/8 (62.5%)
plasma	1wk	ENA-78	8/9 (88.9%)	7/8 (87.5%)
plasma	1wk	Eotaxin-3	1/9 (11.1%)	0/8 (0.0%)
plasma	1wk	GROα	8/9 (88.9%)	7/8 (87.5%)
plasma	1wk	IFN-α2	7/9 (77.8%)	6/8 (75.0%)

Medium	Time	Protein	PGD–	PGD+
plasma	1wk	IL-12p40	4/9 (44.4%)	2/8 (25.0%)
plasma	1wk	IL-12p70	9/9 (100.0%)	7/8 (87.5%)
plasma	1wk	IL-13	7/9 (77.8%)	7/8 (87.5%)
plasma	1wk	IL-16	6/9 (66.7%)	6/8 (75.0%)
plasma	1wk	IL-17A	6/9 (66.7%)	7/8 (87.5%)
plasma	1wk	IL-17E/IL-25	8/9 (88.9%)	7/8 (87.5%)
plasma	1wk	IL-17F	7/9 (77.8%)	6/8 (75.0%)
plasma	1wk	IL-2	7/9 (77.8%)	7/8 (87.5%)
plasma	1wk	IL-20	1/9 (11.1%)	0/8 (0.0%)
plasma	1wk	IL-21	0/9 (0.0%)	0/8 (0.0%)
plasma	1wk	IL-22	5/9 (55.6%)	7/8 (87.5%)
plasma	1wk	IL-23	3/9 (33.3%)	1/8 (12.5%)
plasma	1wk	IL-28A	0/9 (0.0%)	0/8 (0.0%)
plasma	1wk	IL-3	6/9 (66.7%)	3/8 (37.5%)
plasma	1wk	IL-33	2/9 (22.2%)	1/8 (12.5%)
plasma	1wk	IL-5	9/9 (100.0%)	7/8 (87.5%)
plasma	1wk	IL-7	8/9 (88.9%)	8/8 (100.0%)
plasma	1wk	IL-9	4/9 (44.4%)	2/8 (25.0%)
plasma	1wk	LIF	7/9 (77.8%)	5/8 (62.5%)
plasma	1wk	MCP-2	9/9 (100.0%)	7/8 (87.5%)
plasma	1wk	MCP-4	8/9 (88.9%)	4/8 (50.0%)
plasma	1wk	MIP-1α	8/9 (88.9%)	6/8 (75.0%)
plasma	1wk	SCF	5/9 (55.6%)	7/8 (87.5%)
plasma	1wk	TGFα	9/9 (100.0%)	7/8 (87.5%)
plasma	1wk	TNFβ	2/9 (22.2%)	2/8 (25.0%)
plasma	1wk	TPO	4/9 (44.4%)	5/8 (62.5%)
plasma	1wk	TSLP	2/9 (22.2%)	3/8 (37.5%)
plasma	2wk	6CKine	7/7 (100.0%)	6/7 (85.7%)
plasma	2wk	EGF	2/7 (28.6%)	4/7 (57.1%)
plasma	2wk	ENA-78	4/7 (57.1%)	6/7 (85.7%)
plasma	2wk	Eotaxin-3	1/7 (14.3%)	0/7 (0.0%)
plasma	2wk	FLT-3L	7/7 (100.0%)	6/7 (85.7%)
plasma	2wk	GROα	2/7 (28.6%)	5/7 (71.4%)
plasma	2wk	IFN-α2	6/7 (85.7%)	5/7 (71.4%)
plasma	2wk	IFNγ	5/7 (71.4%)	6/7 (85.7%)
plasma	2wk	IL-12p40	3/7 (42.9%)	1/7 (14.3%)
plasma	2wk	IL-12p70	7/7 (100.0%)	6/7 (85.7%)
plasma	2wk	IL-13	5/7 (71.4%)	6/7 (85.7%)
plasma	2wk	IL-16	4/7 (57.1%)	3/7 (42.9%)
plasma	2wk	IL-17A	6/7 (85.7%)	6/7 (85.7%)
plasma	2wk	IL-17E/IL-25	7/7 (100.0%)	4/7 (57.1%)
plasma	2wk	IL-17F	6/7 (85.7%)	6/7 (85.7%)
plasma	2wk	IL-2	5/7 (71.4%)	2/7 (28.6%)
plasma	2wk	IL-20	0/7 (0.0%)	0/7 (0.0%)
plasma	2wk	IL-21	0/7 (0.0%)	0/7 (0.0%)
plasma	2wk	IL-22	4/7 (57.1%)	6/7 (85.7%)
plasma	2wk	IL-23	1/7 (14.3%)	1/7 (14.3%)
plasma	2wk	IL-28A	0/7 (0.0%)	0/7 (0.0%)
plasma	2wk	IL-3	6/7 (85.7%)	4/7 (57.1%)
plasma	2wk	IL-33	0/7 (0.0%)	0/7 (0.0%)
plasma	2wk	IL-9	4/7 (57.1%)	2/7 (28.6%)
plasma	2wk	LIF	3/7 (42.9%)	4/7 (57.1%)

Medium	Time	Protein	PGD–	PGD+
plasma	2wk	MCP-2	7/7 (100.0%)	6/7 (85.7%)
plasma	2wk	MIP-1α	6/7 (85.7%)	5/7 (71.4%)
plasma	2wk	SCF	4/7 (57.1%)	6/7 (85.7%)
plasma	2wk	TGFα	6/7 (85.7%)	6/7 (85.7%)
plasma	2wk	TNFβ	1/7 (14.3%)	0/7 (0.0%)
plasma	2wk	TPO	2/7 (28.6%)	2/7 (28.6%)
plasma	2wk	TSLP	0/7 (0.0%)	2/7 (28.6%)
plasma	2wk	VEGF-A	6/7 (85.7%)	7/7 (100.0%)
BAL	2hr	6CKine	1/8 (12.5%)	2/8 (25.0%)
BAL	2hr	CTACK	6/8 (75.0%)	8/8 (100.0%)
BAL	2hr	EGF	8/8 (100.0%)	6/8 (75.0%)
BAL	2hr	Eotaxin-3	1/8 (12.5%)	1/8 (12.5%)
BAL	2hr	GM-CSF	7/8 (87.5%)	6/8 (75.0%)
BAL	2hr	IFNγ	8/8 (100.0%)	7/8 (87.5%)
BAL	2hr	IL-17E/IL-25	8/8 (100.0%)	7/8 (87.5%)
BAL	2hr	IL-17F	7/8 (87.5%)	7/8 (87.5%)
BAL	2hr	IL-20	0/8 (0.0%)	1/8 (12.5%)
BAL	2hr	IL-21	0/8 (0.0%)	1/8 (12.5%)
BAL	2hr	IL-22	4/8 (50.0%)	6/8 (75.0%)
BAL	2hr	IL-23	3/8 (37.5%)	4/8 (50.0%)
BAL	2hr	IL-27	7/8 (87.5%)	5/8 (62.5%)
BAL	2hr	IL-28A	0/8 (0.0%)	1/8 (12.5%)
BAL	2hr	IL-3	1/8 (12.5%)	1/8 (12.5%)
BAL	2hr	IL-33	2/8 (25.0%)	3/8 (37.5%)
BAL	2hr	IL-4	7/8 (87.5%)	4/8 (50.0%)
BAL	2hr	IL-7	7/8 (87.5%)	6/8 (75.0%)
BAL	2hr	LIF	0/8 (0.0%)	3/8 (37.5%)
BAL	2hr	MCP-2	7/8 (87.5%)	8/8 (100.0%)
BAL	2hr	MCP-4	2/8 (25.0%)	1/8 (12.5%)
BAL	2hr	MIP-1α	7/8 (87.5%)	8/8 (100.0%)
BAL	2hr	PDGF-AB/BB	3/8 (37.5%)	5/8 (62.5%)
BAL	2hr	TNFβ	6/8 (75.0%)	8/8 (100.0%)
BAL	2hr	TPO	0/8 (0.0%)	3/8 (37.5%)
BAL	2hr	TSLP	0/8 (0.0%)	0/8 (0.0%)
BAL	2hr	VEGF-A	8/8 (100.0%)	5/8 (62.5%)

Distributions of pooled protein concentrations at each time point were right-skewed; those following the offset log-transformation $x \mapsto \log(x + 1)$ (henceforth “log-concentrations”) were roughly normal, and these values were used throughout. Research IDs used internally were uniquely coded to capital letters for reporting of results.

We included all calculated observed concentrations as numeric values, including those interpolated and those extrapolated using standard limit curves. Remaining entries were out-of-range (“OOR”, “OOR <”, or “OOR >”) and not calculated from fluorescence intensities (FIs). When the FI was below the smallest dilution on the limit curve, we imputed a concentration value obtained as the smallest value calculated for that protein in that medium, across all cases—and, in the case of plasma, across all time points. Analogously, we imputed the largest calculated value for OOR entries when the FI was above the largest dilution on the limit curve.

Multiplex assay data from perfusate and BAL fluid were pre-processed in the same way as those from plasma. Note that one set of limit curves was provided for each protein in perfusate and in BAL concentration measurements.

Proteins were not excluded *a priori* due to high rates of missingness. Instead, proteins were excluded as

needed by each analysis, as explained below.

Recipient characteristics

Recipient age, sex, and BMI and LAS score at listing were collected. We also determined whether each recipient was on ECMO pre-LTx and post-operatively and whether they received antithymocyte globulin (ATG) treatment. Spirometry results including PaO2, FiO2, and P/F ratio and the presence of bilateral infiltrates on chest radiograph were recorded at 0hr, 24hr, 48hr, and 72hr.

Each recipient was graded for PGD at 0hr, 24hr, 48hr, and 72hr independently by two pulmonologists according to criteria established by the International Heart and Lung Society (Snell et al. 2017). Only patients with a PGD grade of 0 or 3 for the entire 72hr post-transplant period were included in this study and are referred to as PGD– and PGD+, respectively.

Protein concentrations and pairwise associations

We visualized co-concentration patterns using heatmaps of proteins by subjects (perfusate and BAL fluid) or subject–time points (plasma) with row and column dendrogram annotations based on complete-linkage clustering by euclidean distance (Kolde 2019). For each medium, and for plasma at the three time points nearest time of transplant, we rendered heatmaps both for all subjects and separately for PGD– and PGD+ subjects.

We generated triangular correlation heatmaps of proteins with at least one correlate for each set of biological media (Wei et al. 2021). Proteins were ordered according to complete-linkage clustering dendrograms.

To characterize changes in concentrations over time (across all post-LTx time points), we used Kruskal–Wallis tests to identify proteins that had non-constant concentrations over time. Based on these values, we opted for the more restrictive (unadjusted) threshold $p < 0.01$ because the proteins that met this threshold were adequate to characterize trajectories. We then performed a principal components analysis (PCA) of all subject–time points (including pre-LTx) in the space of the log-concentrations of these proteins. We generated a row-principal biplot of subject trajectories along the first two PCs, and we overlaid segments connecting consecutive centroids in order to visualize the average trajectory.

Discrimination of PGD

To complement the PCA biplot with individual and mean trajectories, we constructed a similar biplot with mean trajectories stratified and color-coded by PGD group (Figure M1).

We visually compared the log-concentrations in perfusate and in BAL as jitter plots and in plasma as line plots between the PGD– and PGD+ subgroups, in each case stratified (main text) or faceted (Figures M2 and M3) by protein. Facets were ordered by the unadjusted p-values obtained from Kolmogorov–Smirnov (perfusate and BAL) or multivariate Cramér (Baringhaus and Franz 2004) (plasma, using all time points) tests of their ability to discriminate between the PGD– and PGD+ subgroups. Kolmogorov–Smirnov and Cramér tests required at least one observation from each PGD group. We only plotted proteins with at least three observations in each PGD group.

We also generated another biplot of subject trajectories in plasma, with markers color-coded and trajectories stratified by PGD group. Additionally, we conducted PCA and generated biplots, again color-coding markers by PGD group, on log-concentrations in perfusate and in BAL fluid.

Prediction of PGD

We used logistic regression (LR), random forest (RF), and nearest neighbors (NN) models in a machine learning (ML) workflow to determine whether several sets of predictors could discriminate between PGD– and PGD+ subjects.

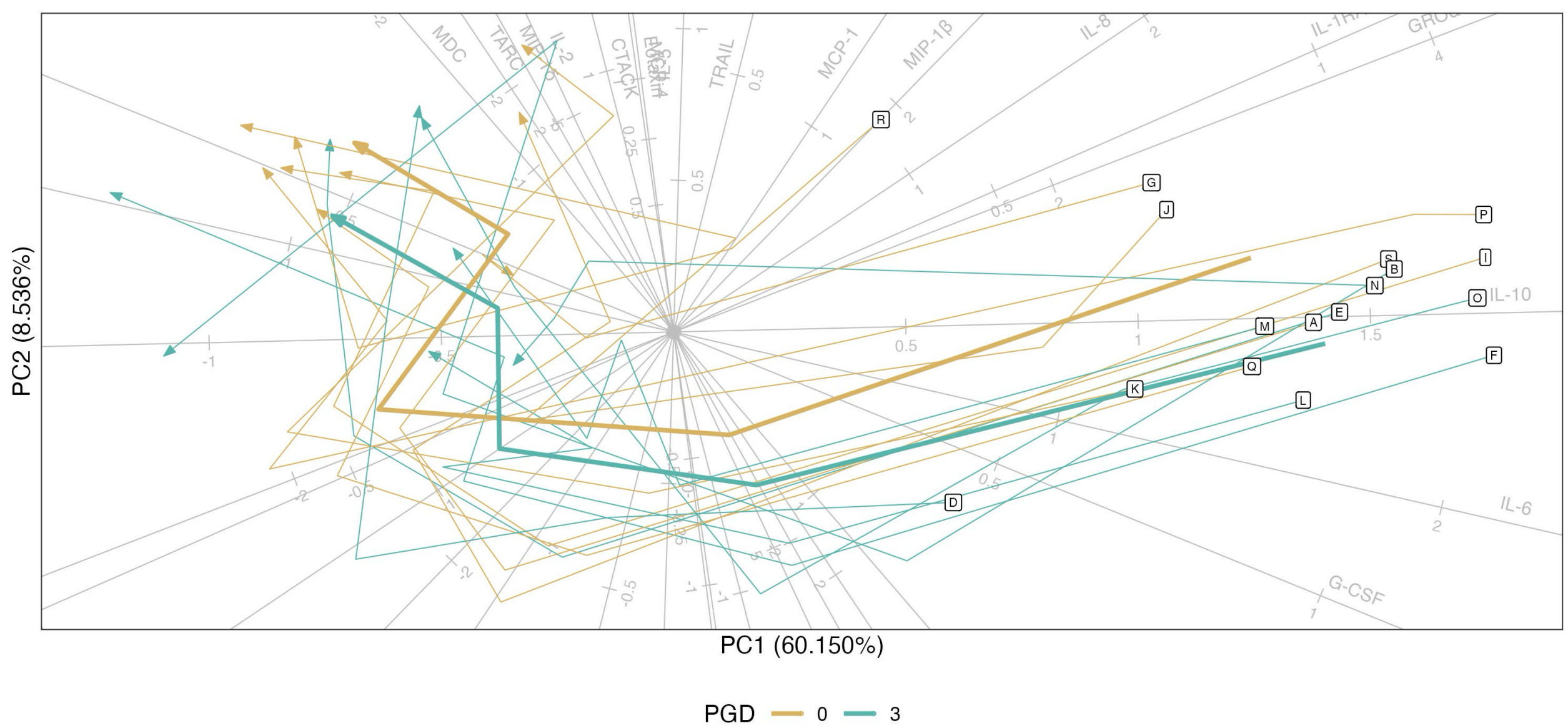

Figure M1: PCA biplot of time-discriminating proteins at all subject–time points, group-averaged by PGD grade (compare to Figure 1C in the main text).

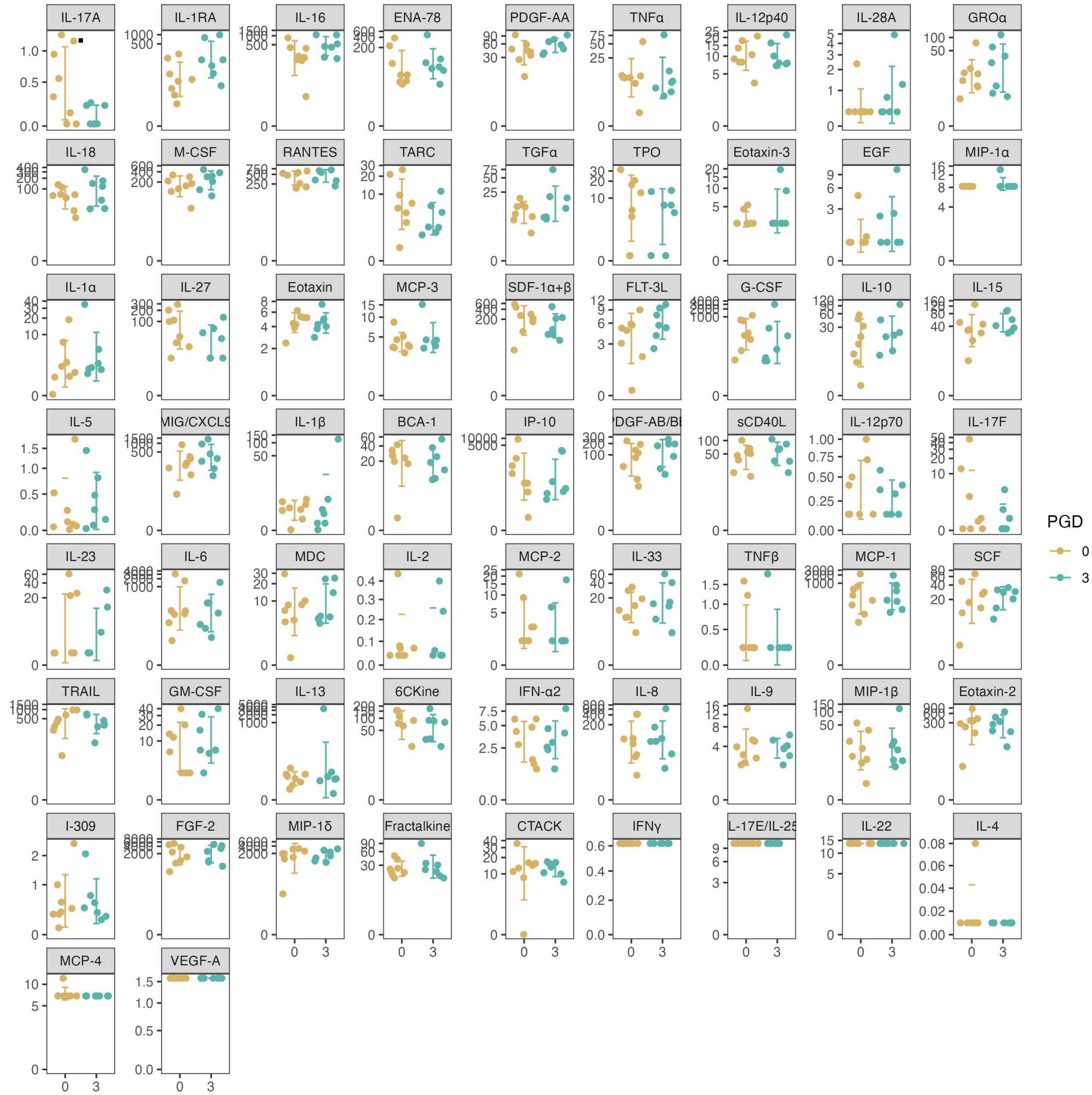

Figure M2: Jitter plots with 1–standard deviation intervals, grouped by PGD grade. The panels are in order of increasing Kolmogorov–Smirnov test unadjusted p-value and marked as in Figure 2 (main text).

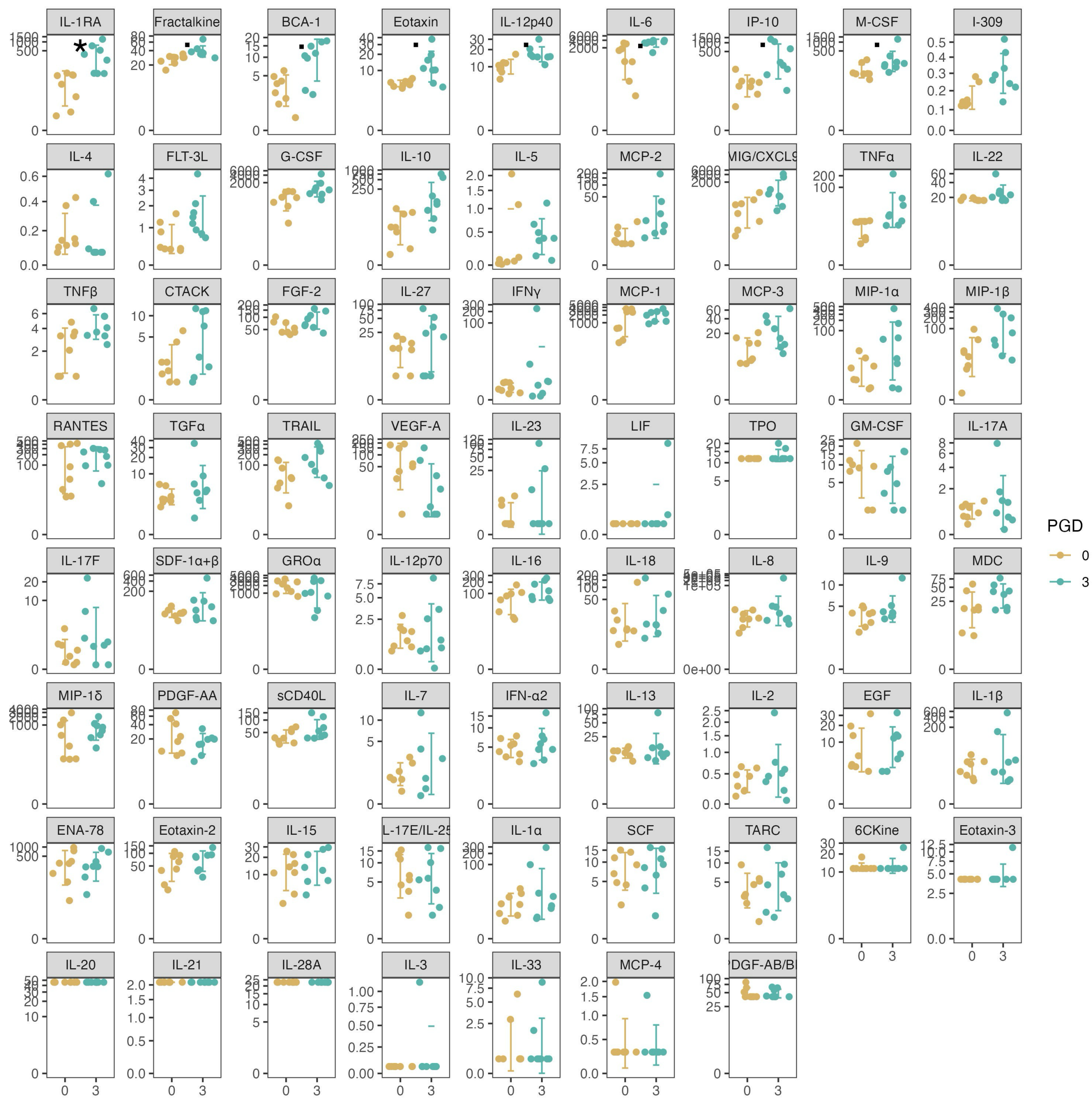

Figure M3: Jitter plots with 1–standard deviation intervals, grouped by PGD grade. The panels are in order of increasing Kolmogorov–Smirnov test unadjusted p-value and marked as in Figure 2 (main text).

We assessed the predictive value of every nonempty subset of 4 data sources: protein levels in the three media (perfusate, BAL fluid, and first three time points of plasma) and the clinical data (which included donor, recipient, and perioperative characteristics). These data sets included the following predictors:

1. Clinical: donor age, donor gender, donor race, storage thermicity, donor smoking history, donor alcohol history, recipient ECMO status pre-Tx and post-op, ATG treatment, recipient LAS score, recipient pre-LTx primary pulmonary diagnosis, recipient age, recipient sex, recipient BMI at listing, and recipient PaO2, FiO2, and P/F ratio for 19 cases.
2. Storage perfusate: multiplex concentrations of 71 proteins at each of 3 time points (pre, 2hr, 24hr) for 16 cases.
3. Post-LTx BAL fluid: multiplex concentrations of 70 proteins (missing IL-7) for 17 cases.
4. Recipient blood plasma: multiplex concentrations of 70 proteins (missing IL-7) for 17 cases.

Combined data were prepared for modeling through a common recipe: All protein concentrations were log-transformed and undefined or infinite values declared missing, and real-valued (including clinical) variables with any missing values were discarded. Categorical (including logical) variables had missing values replaced with the a dummy value for "unknown", while logical variables with complete values were recoded to the integer values 0 and 1. Categorical and logical variables that were constant over our population were discarded. Categorical predictors were expanded into multiple dummy predictors, taking one value of each as the reference value. Finally, any remaining predictors with missing values were discarded.

From the donor, recipient, and perioperative clinical data, organ procurement was discarded due to taking a constant value, and LAS score, recipient BMI, and recipient PaO2, FiO2, and P/F ratio were discarded due to incompleteness. In perfusate, 9 proteins were excluded by the recipe: IFNγ, IL-3, IL-7, IL-22, VEGF-A, IL-20, IL-21, LIF, TSLP. In BAL fluid, 5 proteins were excluded by the recipe: IL-7, IL-20, IL-21, IL-28A, TSLP. Of 213 measurements included from blood plasma over 3 time points, 3 were excluded by the recipe: IL-20 at 2hr, Eotaxin-3 at 24hr, and IL-20 at 24hr.

Models were specified over the following hyperparameter grids:

- LR: penalty λ and regularization mixture α:

$$\{\lambda = 1 \times 10^{-2\ell} : 0 \leq \ell \leq 5\} \times \{\alpha = a/5 : 0 \leq a \leq 5\}$$

- NN: number of neighbors k:

$$\{1 \leq k \leq 9\}$$

- RF: number of trees v and maximum feature split μ, where p is the number of predictors:

$$(\{v = 1\} \cup \{v = 250n : 1 \leq n \leq 8\}) \times \{\mu = \sqrt[3]{p}, \sqrt{p}, p/2, p\}$$

For each model specification, we calculated the area under the receiver operating characteristic curve (AUROC) for leave-one-out cross-validation (LOOCV), stratified by outcome so that each test set comprised one PGD– and one PGD+ case. (Note that the balance of classes in the test set was not known to the model, i.e. the model classified each test case independently and might assign the same class to both cases.) We then repeated this procedure 12 times to ensure diversity in the test sets. We ranked the model specifications by mean AUROC across 12 LOOCVs.

We summarized the means and standard errors of the AUROCs as line–range plots (Figure M4).

We then tabulated the highest AUROC for each combination of data sources and model family (Table M2).

To assess the sensitivity of these optimal performances and present a more realistic expectation of out-of-sample performance, we also tabulated the mean performance of all models whose specifications were within one step on the hyperparameter grid from an optimal specification (Table M3).

We also examined model-based predictor importance measures for the optimized LR and RF models: absolute coefficient estimates for LR and mean decrease in accuracy for RF. Predictors whose maximum absolute coefficient estimates were smaller than 1% of the largest median absolute coefficient estimate were excluded from the LR evaluation, and those whose mean decrease in accuracy was negative were excluded from the RF

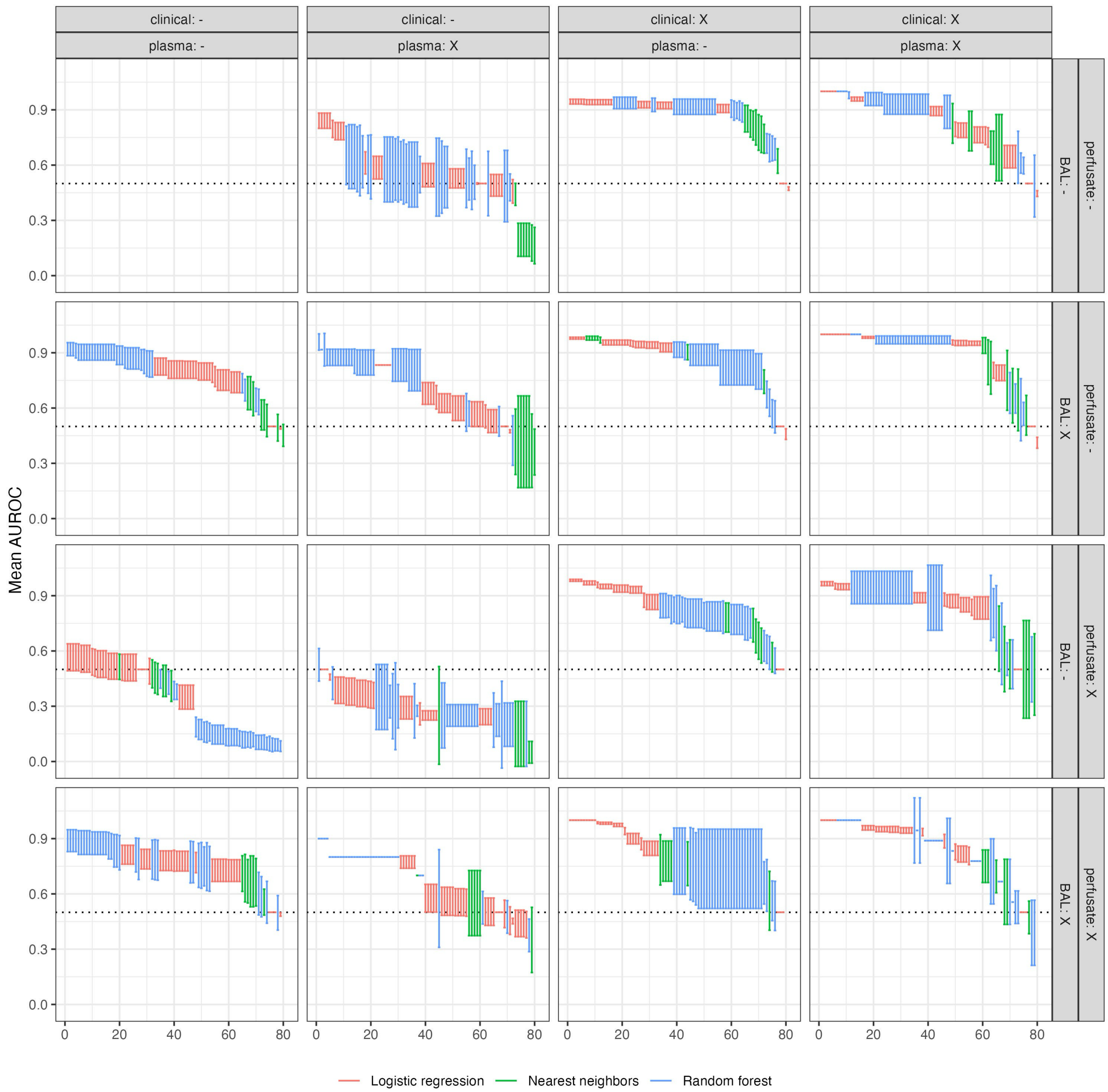

Figure M4: Ranked plots of performances of each model specification across 12 LOOCVs.

				Multiplex				Multiplex and Clinical		
Perfusate	BAL	Plasma	n_0, n_3	LR	NN	RF	n_0, n_3	LR	NN	RF
							10, 9	0.831	0.657	0.718
×			8, 7	0.589	0.438	0.458	8, 7	0.982	0.625	0.574
	×		8, 8	0.906	0.646	0.948	8, 8	1.000	0.906	0.990
		×	9, 8	0.854	0.555	0.651	9, 8	0.771	0.604	0.734
×	×		8, 7	0.756	0.557	0.833	8, 7	0.994	0.738	0.952
×		×	8, 7	0.500	0.339	0.554	8, 7	0.732	0.443	0.607
	×	×	8, 8	0.885	0.479	0.948	8, 8	0.896	0.677	0.938
×	×	×	8, 7	0.768	0.491	0.875	8, 7	0.893	0.613	0.899

Table M2: **Highest predictive performance obtained across parameter grids within each model family and each combination of predictors.** Performance is measured by the area under the receiver operating characteristic curve. BAL: Bronchoalveolar Lavage; LR: Logistic Regression; NN: Nearest Neighbor; RF: Random Forest.

				Multiplex				Multiplex and Clinical		
Perfusate	BAL	Plasma	n_0, n_3	LR	NN	RF	n_0, n_3	LR	NN	RF
							10, 9	0.702	0.646	0.703
×			8, 7	0.579	0.414	0.344	8, 7	0.931	0.622	0.494
	×		8, 8	0.899	0.637	0.876	8, 8	0.952	0.896	0.975
		×	9, 8	0.771	0.551	0.616	9, 8	0.697	0.594	0.690
×	×		8, 7	0.739	0.527	0.820	8, 7	0.873	0.728	0.914
×		×	8, 7	0.411	0.307	0.486	8, 7	0.655	0.423	0.560
	×	×	8, 8	0.839	0.477	0.920	8, 8	0.875	0.646	0.918
×	×	×	8, 7	0.713	0.476	0.841	8, 7	0.849	0.586	0.880

Table M3: **Mean predictive performance across optimal or near-optimal parameter settings within each model family and each combination of predictors.** Performance is measured by the area under the receiver operating characteristic curve and settings that differ in at most 1 parameter by at most 1 grid value are included in the average. BAL: Bronchoalveolar Lavage; LR: Logistic Regression; NN: Nearest Neighbor; RF: Random Forest.

evaluation. Predictor importances (using signed coefficient estimates) were ranked by median importance and plotted on cube root–transformed axes for easier visual comparison.

To test the feasibility of a point-of-care test, we extracted the most important predictors from one data set for inclusion in a reduced model.

For each model family (LR and RF), we computed a cumulative measure of the importance of analyte a as the sum $\bar{I}(a) = \sum_{m \in M*} I_m(a)$ of the importance $I_m(a)$ of a in each model in the collection $M*$ that achieved optimal performance (taken to be zero if m did not use a). We examined the ranking $a_1 \geq \cdot \cdot \cdot \geq a_{71}$ of the analytes by cumulative importance and selected the largest number $3 \leq k \leq 8$ for which $\bar{I}(a_{k+1}) - \bar{I}(a_k) > \bar{I}(a_k) - \bar{I}(a_{k-1})$—that is, up until a drop-off of importance to the next analyte greater than from the previous (Figure M5). This yielded 6 and 8 analytes from the LR (I-309, Fractalkine, IL-12p40, IL-5, FGF-2, PDGF-AA) and RF (IL-1RA, Fractalkine, M-CSF, IL-6, BCA-1, IP-10, IL-12p40, Eotaxin) model families, respectively, with 2 in common.

We pooled the top predictors from the LR and RF models and used LASSO regularization on the full data set to better understand their roles and value in predicting PGD group. Based on the LASSO, we selected a subset as a candidate biomarker panel. We visualized the relationship of the selected predictors to PGD grade using PCA and evaluated the performance and calibration of the logistic regression model using that subset.

While standard diagnostic plots are difficult to interpret for GLMs, those obtained indicated that the reduced model is well-calibrated on most cases, but that a few exceptional cases exert outsized influence (Figure M6). Randomly-generated quantile residuals (Dunn and Smyth 1996) provide a more appropriate set of diagnostics.

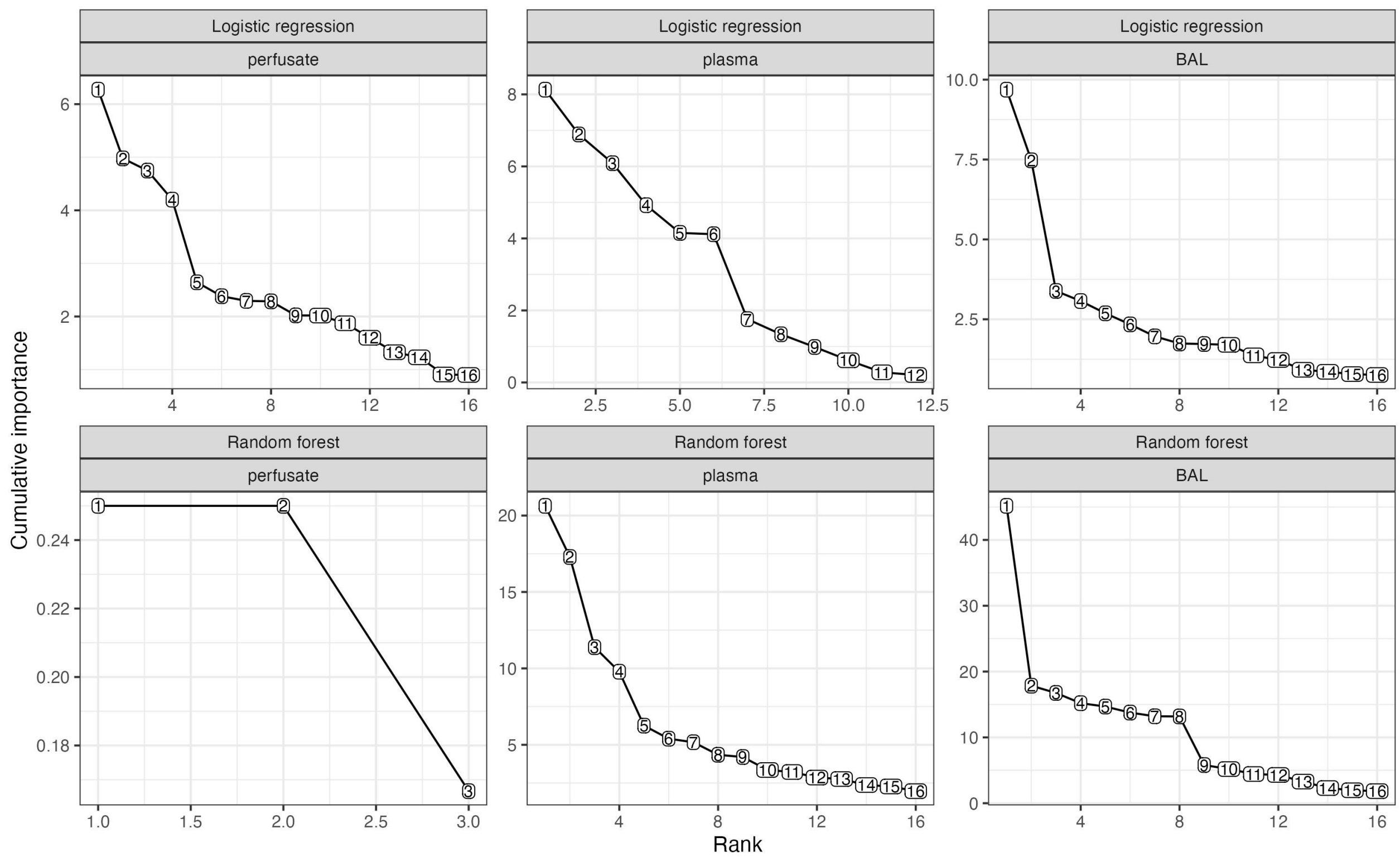

Figure M5: Top 16 cumulative importance measurements $I_C(a)$ for analytes a in each model family, based on optimized models fitted to full data. Cutoffs were imposed at $I_C \leq 5$ (LR) and at $I_C \leq 7$ (RF).

We viewed 6 batches, were slightly skewed left but otherwise indistinguishable from Gaussian, which suggests that the model is a reasonable fit to the data (Figure M7). (The issues identified here are to be expected for such a small set of cases.)

Individual clinical predictors

The pooled clinical variables were more predictive than any single biological source, but the comparison above does not disaggregate the roles of individual donor, recipient, and perioperative factors. While not the focus of our study, as noted by an anonymous reviewer, it is important to note whether and which of these factors might explain the variation in outcomes. Table M4 reports optimized AUROCs for logistic regression models of PGD– versus PGD+ using individual clinical predictors. The values can be compared to those of Table M2.

Table M4: Optimized AUROC for logistic regression models of undetected versus severe primary graft dysfunction on individual clinical predictors.

group	Predictor	AUROC	AUPRC
Donor	Age	0.500	0.759
NA	Gender	0.780	0.885
NA	Racial Cat.	0.500	0.759
NA	Alcohol Hx	0.500	0.759
NA	Smoking Hx	0.576	0.758
Recipient	Age	0.597	0.759
NA	BMI	0.616	0.759
NA	Sex	0.787	0.890
NA	Pre-LTx Dx	0.532	0.759
NA	UNOS LDx Group	0.706	0.860
NA	LAS Score	0.625	0.759
Perioperative	Static Storage	0.722	0.864
NA	Pre-LTx ECMO	0.602	0.770

Robustness checks

In discussion among three authors (JCB, DN, CA), we made three choices regarding the main analysis of multiplex data: First, we chose to include all calculated observed concentrations provided by Eve Technologies, including those extrapolated from outside the limit curves. Second, for discrimination analysis, we chose to impute values where these were missing due to fluorescence intensities being too far outside the limit curve. For values below (above) each limit curve, we imputed the minimum (maximum) calculated observed concentration obtained in the same medium across all cases (and, in the case of plasma, time points). For predictive modeling, we did not impute, and variables with missing (out-of-range) values were excluded. This enabled us to compare some analytes using more cases than for which interpolated values were available with a conservative bias in the resulting test statistics. Third, we chose to exclude data obtained from an earlier batch used to test the assay. This batch included samples from two cases, one PGD+ and one PGD–. While it would have increased our sample sizes significantly, we were not prepared to incorporate batch effects into our analysis.

To test the robustness of our results to these choices, we re-ran the full analysis using three alternative settings, in each case holding other choices fixed: (1) using concentrations without imputations for discrimination analysis and with imputations for predictive modeling; (2) using fluorescence intensities (FIs) rather than calculated observed concentrations; and (3) including data for two additional cases assayed in a different batch.

We compared the results obtained in both robustness checks to those obtained in the main analysis to identify

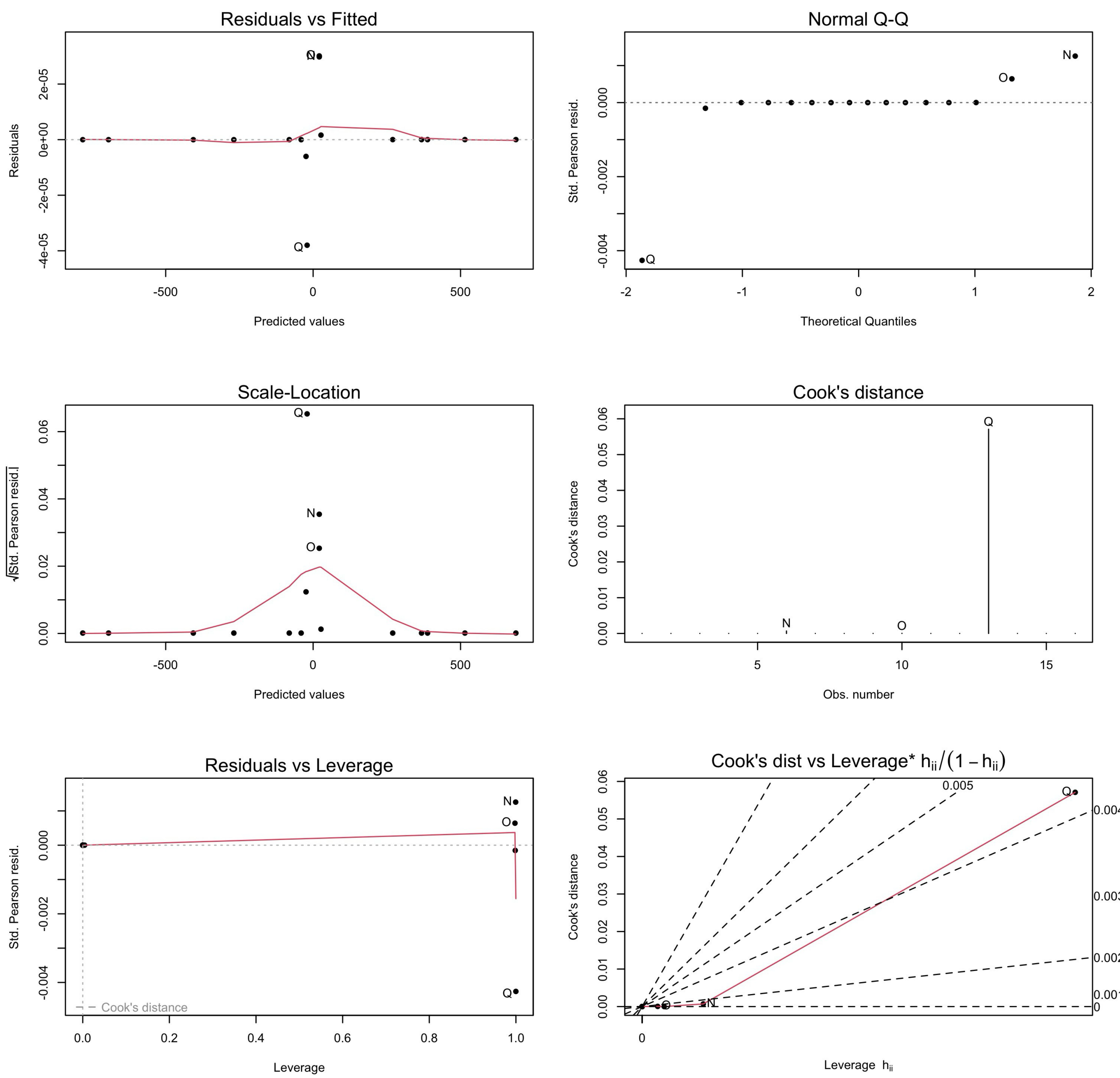

Figure M6: Diagnostic plots for linear regression models provided by ‘plot.lm()‘ in the R stats package; left-to-right, top-to-bottom: (i) residuals versus fitted values, (ii) quantile–quantile normality plot, (iii) root-absolute residuals versus fitted values, (iv) Cook’s distance (versus row index), (v) residuals versus leverage, and (vi) Cook’s distance versus odds-transformed leverage.

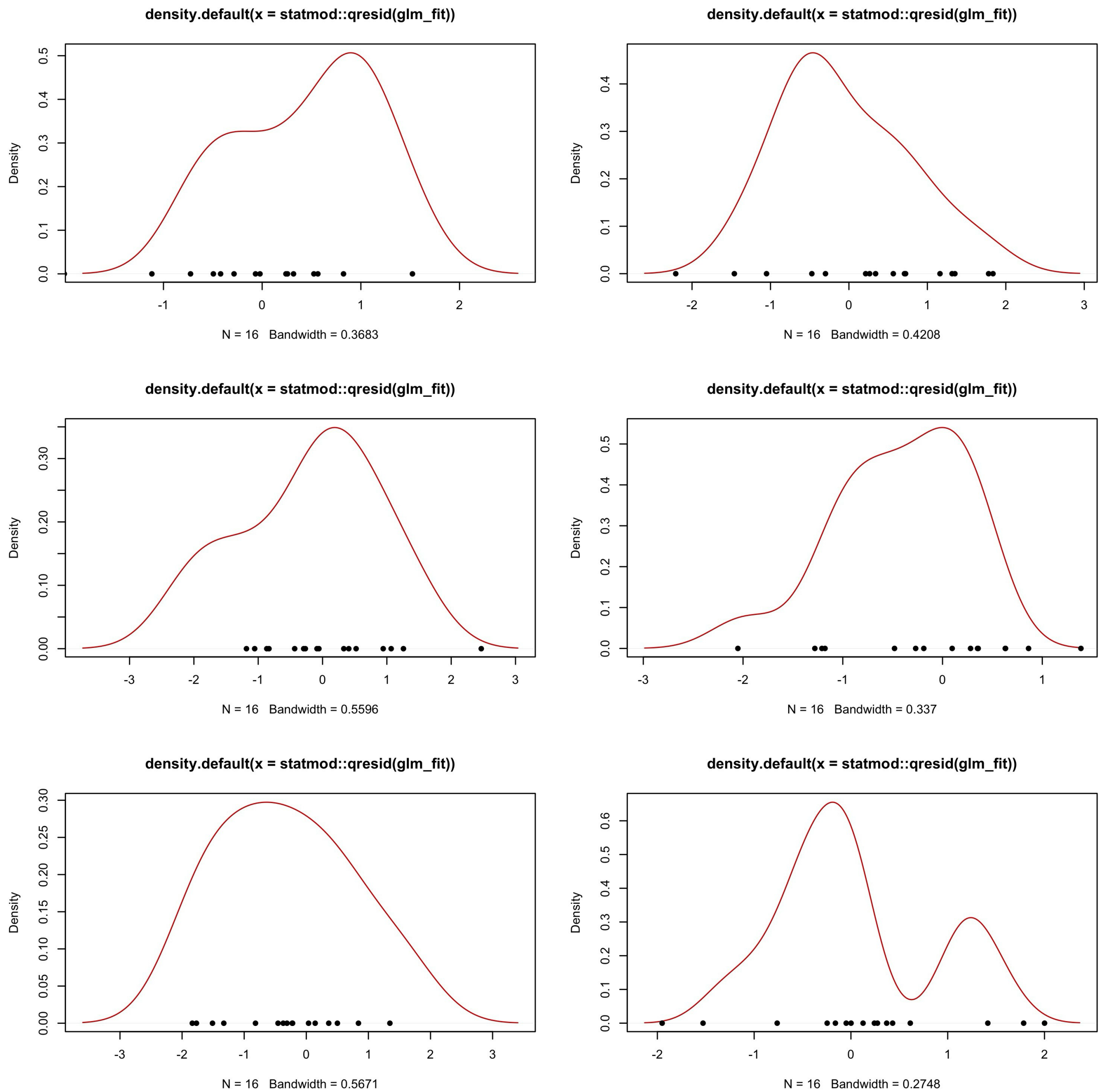

Figure M7: Density plots of 6 batches of randomized quantile residuals. Deviations from normality suggest violations of the exponential distribution assumption underlying logistic regression.

any qualitative differences, and we calculated the differences in optimal performance between corresponding model families and predictor sets.

References

Baringhaus, L., and C. Franz. 2004. "On a New Multivariate Two-Sample Test." *Journal of Multivariate Analysis* 88 (1): 190–206. https://doi.org/10.1016/S0047-259X(03)00079-4.

Dunn, Peter K., and Gordon K. Smyth. 1996. "Randomized Quantile Residuals." *Journal of Computational and Graphical Statistics* 5 (3): 236–44. https://doi.org/10.1080/10618600.1996.10474708.

Kolde, Raivo. 2019. "Pheatmap: Pretty Heatmaps."

Snell, Gregory I., Roger D. Yusen, David Weill, Martin Strueber, Edward Garrity, Anna Reed, Andres Pelaez, et al. 2017. "Report of the ISHLT Working Group on Primary Lung Graft Dysfunction, Part I: Definition and Grading—A 2016 Consensus Group Statement of the International Society for Heart and Lung Transplantation." *The Journal of Heart and Lung Transplantation* 36 (10): 1097–1103. https://doi.org/10.1016/j.healun.2017.07.021.

Wei, Taiyun, Viliam Simko, Michael Levy, Yihui Xie, Yan Jin, Jeff Zemla, Moritz Freidank, Jun Cai, and Tomas Protivinsky. 2021. "Corrplot: Visualization of a Correlation Matrix."
